# Unveiling cascading lag effects of wetland methane emissions: Evidence from Lake Chad in Africa

**DOI:** 10.1126/sciadv.adx9866

**Published:** 2026-07-29

**Authors:** Ruoqi Liu, Geli Zhang, Mengyao Liu, Shushi Peng, Ronald van der A, Michiel van Weele, Oliver Schneising, Jilin Yang, You Wu, Vincent Huijnen, Michael Buchwitz, Chang Fan, Zizhang Zhao, Xiaoxing Zuo, Jinwei Dong, Xiangming Xiao

**Affiliations:** ^1^ College of Land Science and Technology, China Agricultural University, Beijing 100193, China.; ^2^ KNMI, Royal Netherlands Meteorological Institute, De Bilt, Netherlands.; ^3^ Sino-French Institute for Earth System Science, College of Urban and Environmental Sciences, Peking University, Beijing 100871, China.; ^4^ KNMI–NUIST Center for Atmospheric Composition, Nanjing University of Information Science and Technology, Nanjing 210044, China.; ^5^ Institute of Environmental Physics (IUP), University of Bremen FB1, Bremen, Germany.; ^6^ College of Grassland Science and Technology, China Agricultural University, Beijing 100193, China.; ^7^ Institute of Geographic Sciences and Natural Resources Research, Chinese Academy of Sciences, Beijing 100101, China.; ^8^ School of Biological Sciences, University of Oklahoma, Norman, OK 73019, USA.

## Abstract

Wetland methane (CH_4_) emission seasonality is a major yet uncertain component of the global CH_4_ seasonal cycle, with its quantification primarily limited by the challenges in accurately characterizing wetland dynamics and emission processes. Here, we proposed a remote sensing–based framework to analyze the seasonality of tropical wetland CH_4_ emission systems by integrating satellite methane inversions with high-resolution wetland inundation mapping (distinguishing open water and inundated vegetation). Our results reveal a strong seasonal amplitude (4.75 teragrams per year) and pronounced seasonal hysteresis between precipitation and CH_4_ emissions (7 months) in Lake Chad. We demonstrate that the cascading chain of precipitation–wetland inundation–vegetation succession–CH_4_ emissions drives emission seasonality, characterized by a 3-month lag from precipitation to wetland inundation and a subsequent 4-month lag from inundation to peak emissions. These findings provide comprehensive and mechanistically generalizable insights into tropical wetland CH_4_ seasonality and offer critical constraints for improving tropical CH_4_ flux estimates.

## Teaser

Satellite observations reveal sizeable tropical wetland CH_4_ emission seasonality that lags rainfall and inundation patterns.

## INTRODUCTION

Methane (CH_4_) is the second most potent anthropogenic greenhouse gas after CO_2_, accounting for 22 to 34% of the total increase in radiative forcing from greenhouse gases (*1*). Atmospheric methane concentrations (XCH_4_) have been increasing again since 2007, reaching a record growth rate in 2021 (*2*), which has been attributed to factors including increased tropical inundation (*3*), heightened microbial activity in boreal regions (*4*, *5*), and a decrease in tropospheric OH sinks (*6*). The amplified seasonal cycle of XCH_4_ in the subtropics and tropics has been shown to have a substantial impact on annual budget growth, primarily through the synergy between wet-season low OH and peak biogenic methane production (*7*, *8*). Tropical wetlands, which comprise 50 to 70% of global wetland area (*9*, *10*), contribute notably to uncertainties in the global CH_4_ balance due to their large seasonal variability in spatial extent (*11*), especially within seasonally flooded wetlands such as those in Africa (*12*). Climate warming–driven amplification of river flow seasonality in the tropics (*13*, *14*) may further exacerbate the seasonal dynamics of wetland inundation and CH_4_ emissions (*15*, *16*). However, quantifying and attributing the seasonal cycle of CH_4_ emissions in tropical wetlands remains a formidable challenge.

Uncertainties in wetland CH_4_ emissions mainly arise from varied spatial and temporal patterns of tropical wetland extents, the sparse availability of CH_4_ observations, and limited knowledge of the seasonal features of CH_4_ biogeochemistry (*17*, *18*). Current efforts to characterize wetland extent largely rely on coarse satellite observations or model simulations solely driven by climate variables (*19*, *20*). These wetland area estimates typically ignore the contribution of river discharge and lateral flow within wetlands (*9*), although these water inputs dominate seasonal changes of wetland areas in the tropics. Such simplifications often lead to biases in seasonal cycles of CH_4_ emissions derived from wetland process modeling. Now, satellite CH_4_ observations enable basin-scale emission estimates (*21*). However, the spatial distribution and seasonality of wetland CH_4_ emissions still rely heavily on a priori information on wetland emissions, which differs substantially among existing inventories (*22*). Wetland CH_4_ seasonality is governed by complex interactions between the atmospheric environment, wetland dynamics, and microbial activity. Earlier studies have examined dominant environmental controls across different wetland types (*23*, *24*) and emphasized the critical role of lagged effects of precipitation or temperature on CH_4_ emission peaks (*25*, *26*). However, capturing the full seasonal variability of CH_4_ emissions observed by satellites (*21*) requires a more comprehensive and high-resolution spatiotemporal characterization of the factors in the process. The key challenges lie in the precise quantification of the potential cascading chain of major driving factors—spanning precipitation, wetland inundation, vegetation succession, and CH_4_ production and emission—at fine timescales and in the explicit elucidation of the fundamental mechanism operating at each stage.

Recent progress in remote sensing offers a transformative opportunity to improve our understanding of wetland dynamics and CH_4_ emissions. First, the TROPOspheric Monitoring Instrument (TROPOMI), launched in October 2017 onboard the Sentinel-5 Precursor is one of the most advanced sensors, characterized by improved spatial and temporal coverage of CH_4_ observations (near daily, 5.5 km by 7 km after 6 August 2019). Combined with a prior inventory of wetlands, these observations demonstrate the capability to derive monthly wetland CH_4_ emissions at a spatial resolution of ∼30 km from space (*22*, *26*, *27*). Second, emerging optical multispectral imagers like Sentinel-2 provide high spatial resolution (10 m) and a frequent revisit time (5 days), enabling the monitoring of detailed dynamics of wetland extent at monthly scales (*28*).

Here, we examined the seasonal variation in CH_4_ emissions in relation to seasonal inundation over an extensive tropical African wetland, the Lake Chad region, which comprises both seasonally (85%) and permanently (15%) inundated areas (fig. S1). We used the TROPOMI/WFMD v1.8 research product and a divergence method to quantify monthly CH_4_ emissions over the Lake Chad region from 2019 to 2022 on a spatial grid of 0.2° by 0.2°. Using Sentinel-2 observations, we mapped monthly wetland inundation areas at a high spatial resolution (10 m), distinguishing between open water and inundated vegetation (i.e., marshes). These high-resolution maps of seasonal wetland water and vegetation extent facilitate a detailed correlation analysis with CH_4_ emission changes. This analysis provides mechanistic insight into the drivers of wetland CH_4_ emission variability in the Lake Chad region, which include the contributions of highly variable hydrological processes and vegetation dynamics. The analysis further establishes a generalizable framework that captures the cascading chain of biogeochemical processes governing the seasonal dynamics of tropical wetland CH_4_ emissions.

## RESULTS

### Seasonality of CH_4_ emissions and wetland dynamics

**Fig. 1.**
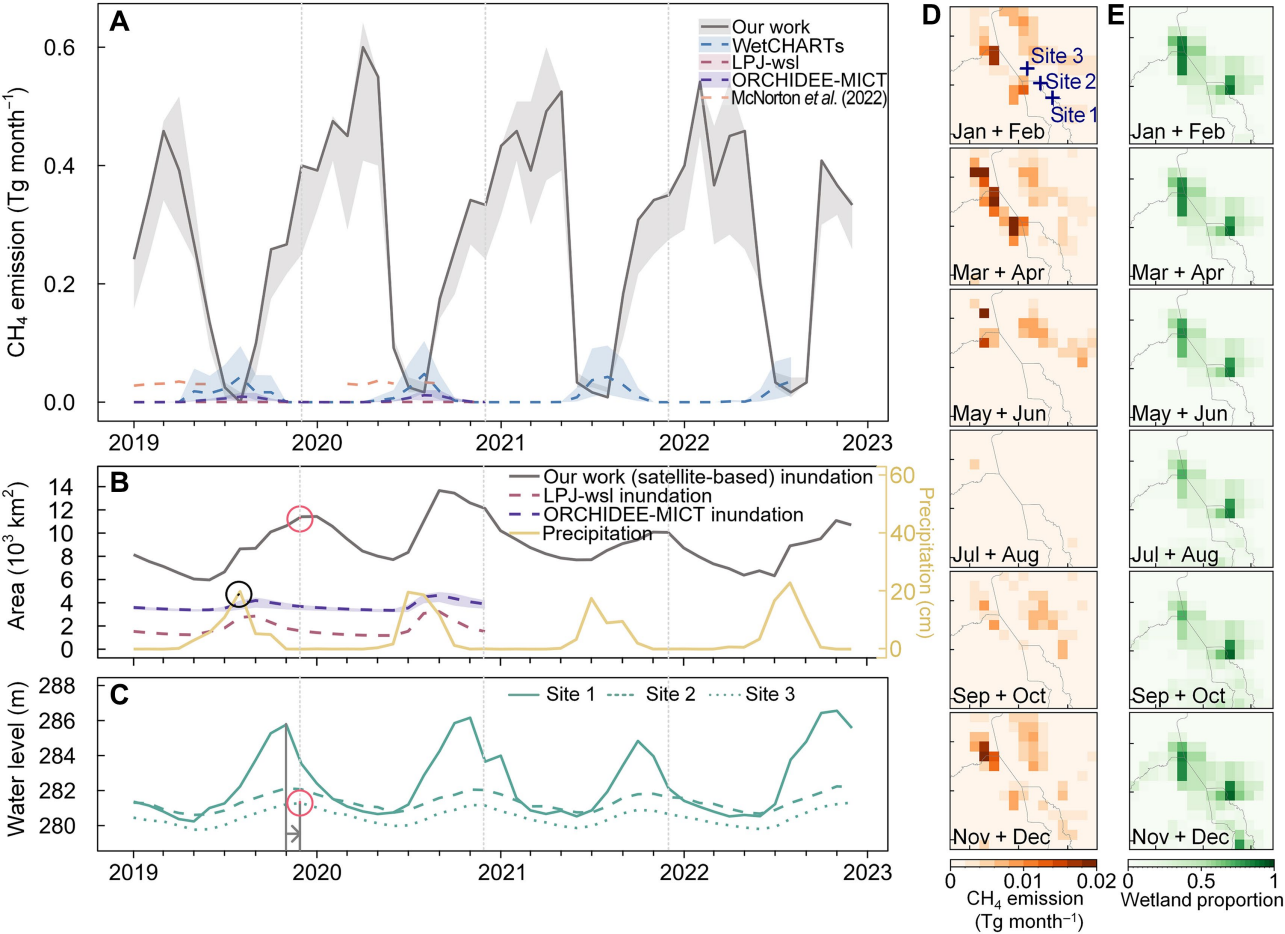
Seasonal dynamics of CH_4_ emissions and wetland inundation across the Lake Chad region. (**A**) Time series of monthly CH_4_ emissions (teragrams per month) derived from TROPOMI, ORCHIDEE-MICT, LPJ-wsl, the ensemble of WetCHARTs v1.3.3, and McNorton *et al.* (*61*). (**B**) Time series of monthly inundation extent from this study, process-based models (LPJ-wsl and ORCHIDEE-MICT), and precipitation from the Global Precipitation Measurement IMERG Final version 07. (**C**) Time series of monthly water levels measured at site 1 (14.71°E, 12.72°N), site 2 (14.45°E, 13.03°N) from DAHITI, and site 3 (13.33°E, 14.17°N) from Hydroweb (https://hydroweb.next.theia-land.fr/). Site locations are denoted in the first panel of (D). (**D** and **E**) Spatial patterns of bimonthly average CH_4_ emissions and wetland inundation fractions in 2019 at a 0.2° resolution. The wetland inundation area represents the sum of open water and inundated vegetation, aggregated from 10 m to the 0.2° grid scale. In (B) and (C), circles indicate the timing of peak methane emissions, precipitation, and local water level. The gray arrow represents the time lag between the peak in local water level and the peak in upstream river water level.

By integrating TROPOMI observations and Sentinel-2 imagery, we quantified monthly CH_4_ emissions and wetland inundation areas (explicitly distinguishing between open water and inundated vegetation) with unprecedented spatial resolution over the Lake Chad region. Both CH_4_ emissions and wetland vegetation-water components exhibit strong seasonal variations (Fig. 1). Over the 2019–2022 period, CH_4_ emissions exhibited pronounced seasonality, peaking in winter (December to February) and spring (March to May) at ∼3.74 to 5.43 Tg year^−1^ (accounting for 74% of annual emissions; Fig. 1, A and D). Conversely, emissions dropped to 1.40 to 1.93 Tg year^−1^ in summer (June to August) and autumn (September to November), forming a distinct “V”-shaped annual cycle characterized by a midyear trough. Despite interannual variability, this considerable seasonal pattern persisted across all years. We observed seasonal wetland inundation variations between the maximum in December (13,877 km^2^ on average, except in 2020) and the minimum in June/July (6927 km^2^ on average), representing a 50% reduction in area at a time that coincides with peak precipitation events (Fig. 1, B and E), suggesting a substantial phase lag relative to meteorological forcing. The distinct seasonal patterns and spatial distributions of open water and inundated vegetation, the two primary wetland components, necessitate their separate consideration in wetland CH_4_ flux analyses (fig. S2).

Our results reveal a strong seasonal amplitude in CH_4_ emissions (4.75 Tg year^−1^) that is largely absent in current widely used process-based models [WetCHARTs (*29*), LPJ-wsl (*12*), and ORCHIDEE-MICT (*6*), which estimate only 0.48 Tg year^−1^ or near zero amplitudes; Fig. 1A]. This discrepancy remains evident after accounting for permanent open water contributions [∼0.20 Tg year^−1^ from diffusion and ebullition; (*30*)], which are minor in tropical vegetated wetlands (*31*) and typically excluded from model calculations (*19*, *20*). These mismatches in seasonal emission magnitudes align with findings for other tropical wetland CH_4_ hot spots, such as the Sudd and Okavango Delta in Africa and the Pantanal in South America (*21*, *22*, *26*). Such underestimation likely arises from the coarse spatial resolution (≥0.5°) of process-based models, which fail to resolve fragmented wetland dynamics (Fig. 1, B and C). In these regions, unrepresented small tropical wetland areas (<0.1 km^2^) can disproportionately contribute to total wetland CH_4_ emissions (*32*).

### Hysteresis of CH_4_ emissions

**Fig. 2.**
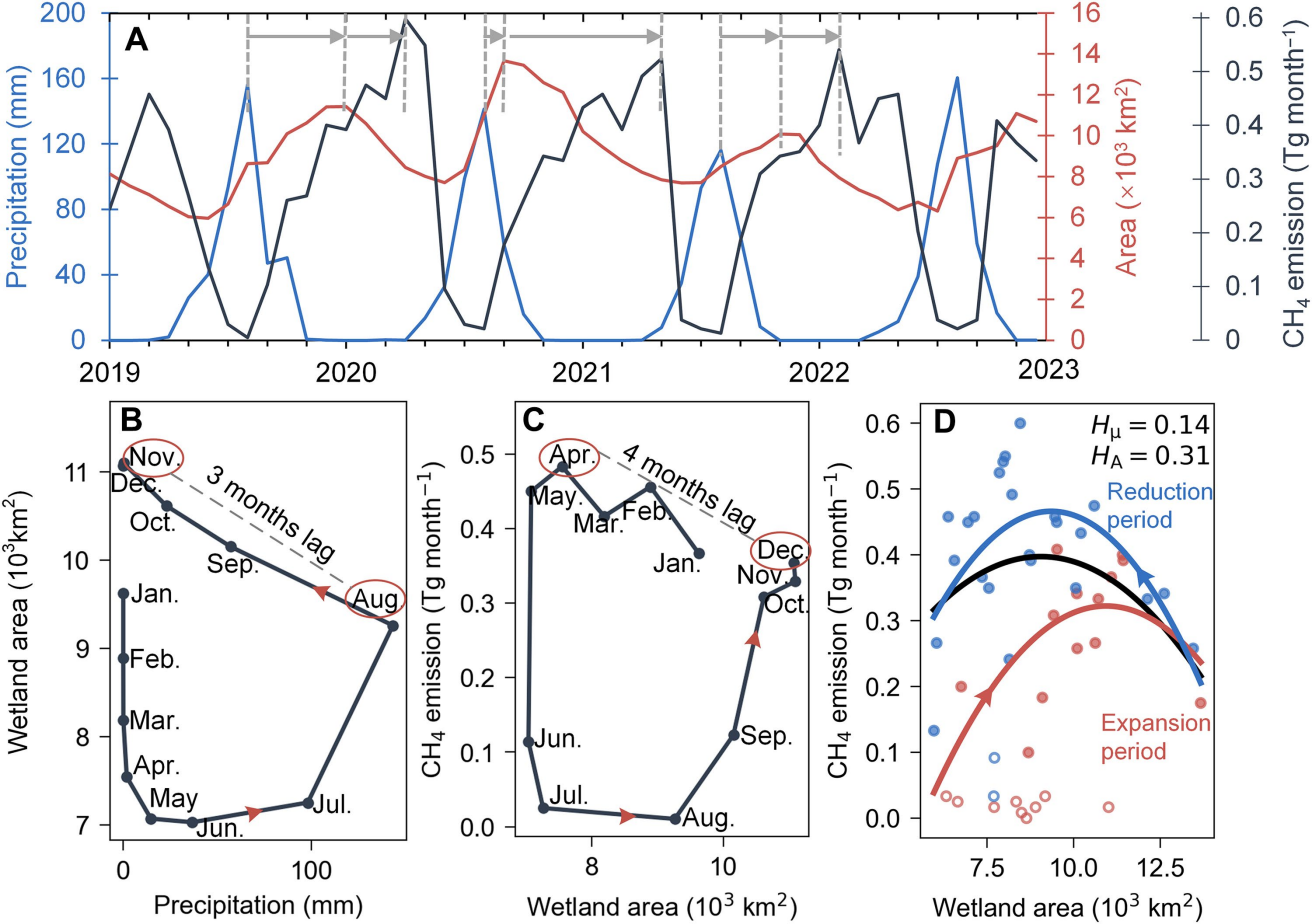
Temporal hysteresis among CH_4_ emissions, wetland inundation areas, and precipitation. (**A**) Time series of monthly precipitation (millimeters), water areas (10^3^ km^2^), and CH_4_ emissions (teragrams per month) during 2019–2022. Dashed lines denote peaks of precipitation, water areas, and CH_4_ emissions; arrows indicate the phase lags between precipitation–wetland inundation area and wetland inundation area–CH_4_ emissions. (**B**) Hysteretic relationship between precipitation and wetland inundation area based on 4-year monthly averages. (**C**) As in (B), but for the relationship between wetland inundation area and CH_4_ emissions. (**D**) Functional dependencies of CH_4_ emissions on wetland inundation area (lines) derived from 4-year monthly estimates (dots). Open circles represent data affected by poor-quality satellite observations and are excluded from the polynomial regression (Materials and Methods). Results corresponding to the expansion (earlier), reduction (later), and complete period of the wetland inundation area dynamics are colored in red, blue, and black, respectively. $H_{A}$ and *H*_μ_ denote the normalized area of seasonal CH_4_ emission hysteresis (normalized area enclosed by the blue and red lines) and the mean seasonal CH_4_ emission hysteresis calculated in the 4 years, respectively.

In addition to a strong seasonal amplitude, our analysis reveals that wetland inundation dynamics exhibit pronounced hysteresis in their relationship with CH_4_ emissions (Fig. 2A). Figure 2C demonstrates a distinct counterclockwise loop in the phase space of monthly CH_4_ emissions versus wetland inundation areas, with emission peaks lagging inundation maxima by 4 months. This pattern matches the results of temporal cross-correlation analysis between monthly CH_4_ emissions and wetland inundation areas (fig. S3, A and C).

The CH_4_-wetland relationship exhibits pronounced time-dependent variations between wetland expansion [earlier period; coefficient of determination (*R*^2^) = 0.40, *P* = 0.1, *n* = 13] and reduction (later period; *R*^2^ = 0.35, *P* = 0.01, *n* = 24) phases (Fig. 2D). We quantified this intraseasonal dependence using two hysteresis metrics, $H_{A}$ and *H*_μ_, where positive values indicate enhanced CH_4_ emissions during the reduction phase of wetland inundation (Materials and Methods). The result showed substantial seasonal hysteresis between CH_4_ emissions and wetland inundation, with higher emissions during the reduction phase (1.68 Tg year^−1^). Models assuming a seasonally invariant wetland inundation area dependence would neglect this seasonal CH_4_ hysteresis, thereby introducing large biases into CH_4_ estimates: an overestimation (+8.4%) during wetland expansion phases, an underestimation (−23.2%) during reduction phases, and an overall −12.7% bias across the entire annual cycle (black lines, Fig. 2D). These hysteretic patterns demonstrate that representing CH_4_ emissions as a static function of wetland extent is fundamentally inadequate.

Process-based models fundamentally fail to capture this hysteresis of CH_4_ emissions, primarily because their simulated seasonality is mechanistically tied to local precipitation (Fig. 1, A and B). For example, the ORCHIDEE-MICT and LPJ-wsl models implement TOPMODEL-based inundation schemes (*6*) that directly link wetland extent to precipitation inputs, inherently overlooking the observed hysteresis between precipitation and CH_4_ emissions (*17*, *19*). This creates a critical mismatch: While models predict peak emissions in spring/summer coincident with maximum precipitation, our Sentinel-2 and TROPOMI observations reveal minimal emissions and wetland areas during the same period. Crucially, we identified a statistically significant 7-month lag between CH_4_ emission peaks and precipitation peaks (Fig. 2, B and C; fig. S3D; and table S1), a duration substantially longer than the 0 to 2 months reported previously (*17*, *19*).

### Cascading lag effects within the “precipitation–wetland inundation–vegetation succession–CH_4_ emissions” chain

**Fig. 3.**
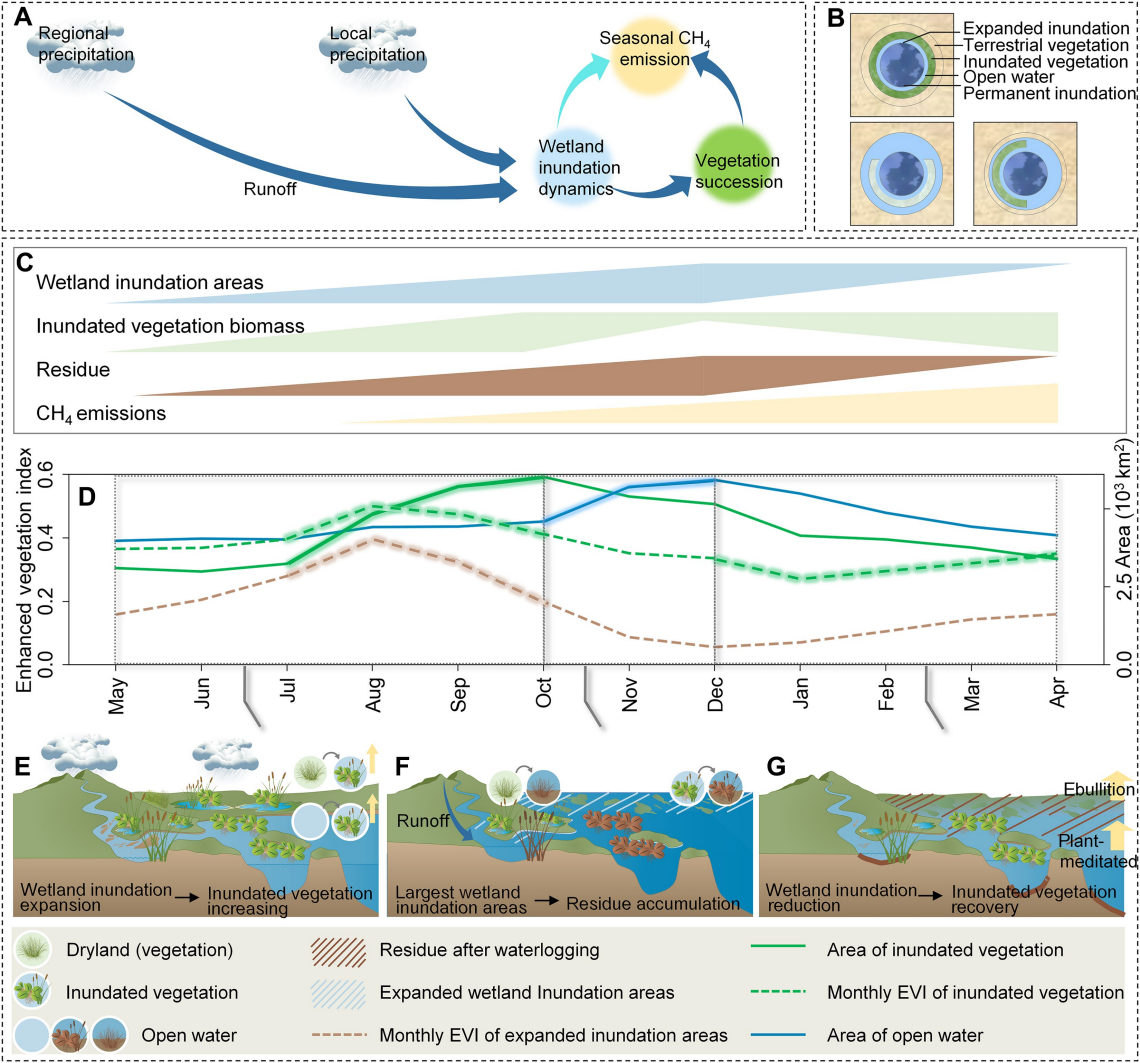
Schematic illustration of the cascading lag effects within the precipitation–wetland inundation–vegetation succession–CH_4_ emission chain. (**A**) Integrated atmosphere-water-vegetation interactions. (**B**) Dynamics of wetland water and vegetation components corresponding to the three key transition periods detailed in (E) to (G). Each ring (not a full circle) represents a distinct land cover type. (**C**) Conceptual diagram of the integrated dynamics governing the tropical wetland CH_4_ emission system. (**D**) Four-year monthly averages of open water area, inundated vegetation area, and EVI. EVI serves as a proxy for vegetation productivity; higher EVI values correspond to increased substrate availability after submergence. The dynamics indicated by the highlighted dashed and solid lines represent the main drivers of CH_4_ emission seasonality at each stage. (**E**) Initial wetland expansion triggered by local precipitation, which increases inundated vegetation area and initial CH_4_ emissions. (**F**) Continued inundation expansion triggered by lagged runoff inflow, leading to the submergence of vegetation and terrestrial surfaces. (**G**) Accumulation of abundant substrate in the anaerobic environment, leading to further increased CH_4_ emissions via ebullition and plant-mediated transport. [(A) and (E) to (G)] Images from the Integration and Application Network/University of Maryland Center for Environmental Science Symbol and Image Libraries (*86*), with required attributions to T. Saxby, Integration and Application Network (ian.umces.edu/media-library), and J. Hawkey, Integration and Application Network (ian.umces.edu/media-library). All images are licensed under a Creative Commons Attribution-ShareAlike International License 4.0 (CC BY-SA 4.0; https://creativecommons.org/licenses/by-sa/4.0/).

The observed 7-month lag emerges from coupled hydrologic-ecologic dynamics, where seasonal wetland water-vegetation interactions modulate methanogenesis. Remote sensing observations trace this cascade: (i) Precipitation initiates riverine flooding, (ii) expanding wetland inundation drives vegetation succession, and (iii) subsequent substrate accumulation and transport facilitate CH_4_ release, forming a cascading lag chain defined as precipitation–wetland inundation–vegetation succession–CH_4_ emissions (Fig. 3, A to C).

#### Lag effects from precipitation to wetland inundation

Initial wetland expansion in low-lying areas is directly triggered by local precipitation (Fig. 3, D and E). However, wetland inundation persists in its expansion after peak rainfall as river discharge from upstream areas gradually redistributes water across the basin (Fig. 3, D and F). This results in a 3-month lag between peak precipitation and peak wetland inundation. Permanent inundation maintains stable surface areas year-round, whereas seasonal inundation, primarily fed by southern river inflows, exhibited a progressive northward expansion from the southern lake margin. These transient floodwaters persisted for a short period, typically 2 to 4 months (fig. S4).

#### Lag effects from wetland inundation to vegetation succession

As local precipitation and inundation areas increase, they initially promote concurrent vegetation growth across hydrological gradients, spanning from expanding inundation zones (newly inundated vegetation; Fig. 3, D and E) to adjacent drylands (terrestrial vegetation; fig. S5). Subsequent large-scale runoff gradually converts land and vegetated areas to open water, submerging existing inundated and terrestrial vegetation (highlighted blue line in Fig. 3D and fig. S5). At the time of peak inundation, abundant substrates are produced from the residues of previously submerged vegetation and accumulate in the anaerobic environment. The relatively higher enhanced vegetation index (EVI) values of the expanded inundation areas during the expansion period suggest increased substrate availability after submergence (highlighted brown line in Fig. 3D). As inundation areas recede, the rebound in EVI of the monthly inundated vegetation areas indicates vegetation recovery in previously inundated zones (Fig. 3G, highlighted light green dashed line in Fig. 3D). This vegetation succession-induced hysteresis results in sustained and nearly doubled CH_4_ emissions during the reduction phase of inundation (Fig. 2D).

#### Lag effects from wetland inundation and vegetation succession to CH_4_ emissions

In seasonally flooded inland wetlands, hydrologic controls (inundation duration/frequency) and vegetation dynamics govern CH_4_ production and emissions. Flooding regimes establish anaerobic conditions for methanogenesis, while inundated vegetation area and phenology regulate both substrate supply and gaseous transport pathways (*33*). Before the rainy season, CH_4_ emissions remain minimal, limited to permanent water bodies and sparse inundated vegetation (Figs. 2C and 3E). The onset of inundation does not immediately trigger CH_4_ release due to the delayed development of plant-mediated CH_4_ transport pathways. These pathways become effective only after sufficient biomass has developed and aerenchyma tissues are well established in the inundated vegetation (*23*). This lag is evidenced by the August peak in EVI values for inundated vegetation, which lags the inundated vegetation area expansion (highlighted green dashed and solid line in Fig. 3D). As the inundation reaches its peak, substantial CH_4_ is produced because of the abundant residues of previous vegetation (Fig. 3F), which serve as substrates for methanogenesis (*34*). The expansion of inundated areas leads to the short-term accumulation of dissolved CH_4_, where increased water depth suppresses ebullition. As water levels begin to recede following peak inundation, CH_4_ emissions continue to increase, driven by previously accumulated CH_4_ in the deep water escaping to the atmosphere via the aerenchyma tissue of recovered inundated vegetation (*30*, *35*) as well as via bubbling and diffusion (Fig. 3G, highlighted light green dashed line in Fig. 3D). The emission rate accelerates as water recedes toward the optimum water depth due to lower pressure and shorter transport distance, contrasting with deep water where CH_4_ accumulates and oxidizes (*23*, *36*). Hence, the emissions peak after the maximum wetland inundation extent due to the combined effect of inundation dynamics and vegetation succession.

Approximately 65% of the Lake Chad region is seasonally inundated, experiencing this cascading hydrological-ecological process (Fig. 1B), which leads to strong seasonal fluctuations in CH_4_ emissions. In contrast, the remaining permanently inundated areas contribute near-continuous fluxes due to stable anaerobic conditions and warm temperatures (*34*, *37*); their emission magnitudes show limited seasonal variation (*36*). Therefore, the transitional zones, specifically the shorelines largely composed of marshes, which experience alternating dry-wet shifts and vegetation succession, dominate the seasonality of CH_4_ emissions in the region as their dynamic biogeochemical conditions create pulsed CH_4_ release patterns.

## DISCUSSION

Our study explicitly documented cascading lag effects of tropical wetland methane emissions (precipitation–wetland inundation–vegetation succession–CH_4_ emission) using high-resolution remote sensing data. While previous studies have identified the lag effects of temperature on wetland CH_4_ emissions, these findings rely primarily on in situ observations and are limited in their ability to represent dynamic processes at regional scales (*25*, *38*). The lagged response of wetland inundation and CH_4_ emissions to precipitation has been reported for other tropical regions, such as the Sudd and Pantanal (*21*, *39*, *40*); however, standard bottom-up CH_4_ emission estimates often overlook the internal heterogeneity of wetland inundation areas and miss the critical lag in emissions that occurs after inundation. By leveraging unprecedented temporal (monthly) and spatial (10 m) resolution remote sensing data, we quantified the complex hysteresis dynamics of this system. Such detailed information enables us to elucidate the underlying biogeochemical processes and mechanisms of the cascading lag effects. These key processes were largely overlooked in previous studies that relied on seasonal-scale analyses, coarse spatial and temporal resolution of wetland datasets (*26*), or indirect proxies like water level/river height (*22*, *41*).

The multistage cascade effects identified in this study provide critical implications for the improvement of process-based models. First, during the expansion phase of wetland inundation, upstream river discharge induces a 3-month lag from precipitation to wetland inundation, effectively submerging dryland and inundated vegetation. Second, as the wetland inundation area recedes, a vegetation succession process, characterized by the reemergence of inundated vegetation combined with the legacy of substrate accumulated in the previous phase, results in a 4-month lag from peak inundation to peak CH_4_ emissions. Based on these findings, we took the ORCHIDEE model as an example and conducted recalibrated simulations by incorporating the identified cascading lag mechanism and our monthly wetland areas. We implemented two key mechanistic improvements in the ORCHIDEE model: (i) substrate accumulation from submerged vegetation during the expansion phase of wetland inundation and (ii) enhanced vascular transport by newly exposed plants during the reduction phase of wetland inundation (see details in the Supplementary Materials). These explicit constraints demonstrably improve CH_4_ cycling representations in the model and result in tropical CH_4_ estimates in strong alignment with satellite observations (root mean square error of 0.14) in terms of both seasonal magnitude and phase (fig. S6). Previous studies pointed out that process-level representations of wetland CH_4_ emissions must account for seasonal variations in the substrate availability for CH_4_ production, methanotrophy in the rhizosphere, and plant-mediated CH_4_ transport (*33*, *42*, *43*), which are related to vegetation functional types (*22*, *44*). The integrated effects of multistage cascade processes on CH_4_ emission seasonality are often nearly an order of magnitude larger than the effects of an individual process (*23*, *24*)*.* However, these processes remain poorly represented in current tropical wetland CH_4_ models. For example, the baseline ORCHIDEE model does not explicitly account for substrate accumulation and depletion effects and represents the transport capacity of plant aerenchyma as a constant, without seasonal variation, whereas WetCHARTs and LPJ-wsl further simplify methane production and release processes by excluding explicit transport pathways and substrate dynamics (*33*, *45*). Therefore, resolving the uncertainty in wetland extent and seasonal variation remains a high priority for refining bottom-up CH_4_ estimates as these are essential for tracing vegetation succession and detecting hysteresis effects on the seasonality of CH_4_ emissions.

Our study framework of multistage cascading effects is applicable to other tropical wetland CH_4_ emission seasonality. While the specific lag times are basin dependent, the cascading chain “precipitation–inundation–vegetation succession–CH_4_ emissions” is a fundamental characteristic of seasonally flooded tropical wetlands. For example, the Sudd, Pantanal, and Okavango Delta are characterized by high hydrological connectivity to river networks and dynamic inundation regulated by river discharge. Previous studies using process-based models and satellite-derived CH_4_ estimates have reported lagged responses of wetland extent and CH_4_ emissions to precipitation in these basins (*21*, *26*, *39*). Specifically, Gerlein-Safdi *et al.* (*39*) identified an ∼2-month lag between precipitation and inundation in the Sudd and Pantanal, which compares to the 3-month hydrological lag that we identified in the Lake Chad region. This difference in lag magnitude is shaped by variations in geographic location, terrain slope, hydrological processes, and vegetation composition across wetlands. Our study demonstrates that advanced methodology and high-resolution satellite observations enable the characterization of monthly inundation and vegetation extent at a higher spatial scale, facilitating a comprehensive illustration of the cascading effects on CH_4_ seasonality in tropical wetlands.

While submonthly variations may exist, a monthly resolution is sufficient to resolve the long-term cascade process (∼7 months) and associated hysteresis, as well as the gradual wetland water variations and vegetation transitions (fig. S5B). Previous studies also showed that, in pantropical wetland areas, weekly surface water extent exhibits peak-trough transitions occurring over monthly intervals (*46*); similarly, aquatic vegetation remains in seed dormancy under several months of deep flooding and is (re)established through germination following water recession with reaeration and light. Field and satellite observations indicate that these vegetation responses rely on water level, temperature, and turbidity and occur at weekly-to-monthly scales (∼4 to 10 weeks) (*47*, *48*).

For further studies in other tropical wetland CH_4_ systems, a combination of high-resolution data is promising for capturing finer-scale variations. Incorporating Synthetic Aperture Radar (SAR) data as a complement to Sentinel-2–based approaches will better capture the full extent of seasonal wetlands in the tropics, particularly in cloud-prone regions. SAR C-band (e.g., Sentinel-1) can penetrate clouds and detect surface water under certain vegetation canopies via double-bounce signals. Nevertheless, there are notable challenges in using Sentinel-1 C-band exclusively to map inundated vegetation transitions. Specifically, C-band often exhibits signal ambiguity between sparse or short vegetation and open water because the typical double-bounce signature is replaced by the single reflection of surface water in these areas (*28*). Furthermore, C-band signals are strongly attenuated by dense vegetation, limiting the detection of surface water under closed canopies (*49*). L-band SAR data (e.g., ALOS-2 PALSAR-2), with its longer wavelength, penetrates the canopy more effectively and can detect seasonal vegetation structure in densely vegetated wetland areas (*39*, *50*). The Sentinel-5 mission will complement TROPOMI with the SWIR band, supporting proxy-CO_2_ retrievals of XCH_4_ with better spatial coverage and accuracy (*51*). Furthermore, atmospheric transport modeling, such as Weather Research and Forecasting Greenhouse Gas Model (WRF-GHG), incorporating improved prior inventories, can provide more detailed and robust estimates of wetland CH_4_ emission seasonality under various environmental conditions.

In conclusion, this study identified a pronounced CH_4_ emission seasonality in the Lake Chad region, with a substantial seasonal amplitude comparable to the total annual CH_4_ emissions from oil and gas production in the Permian Basin (*52*, *53*), a well-studied region of a similar size. Moreover, we precisely quantified the seasonal hysteresis dynamics of CH_4_ emissions and elucidated the underlying multistage cascading process, spanning precipitation, wetland inundation, and vegetation succession. The explicit mechanistic explanation of wetland CH_4_ emissions improved CH_4_ flux estimates within process-based models. Furthermore, our framework of the multistage cascading effects in the Lake Chad region is mechanistically generalizable to other tropical wetlands. With the warming-driven increase in the seasonal amplitude of lake surface extent and river flow in the tropics (*13*, *14*), both wetland inundation and vegetation succession are expected to have stronger responses and substantially affect CH_4_ emission seasonality. Our findings underscore that incorporating these cascading processes into Earth system models is essential for reducing uncertainties in understanding CH_4_ flux in future climate changes.

## MATERIALS AND METHODS

### Study area

The Lake Chad region (24°29′N to 30°04′N, 113°34′E to 118°28′E), spanning parts of Chad, Cameroon, Niger, and Nigeria, covers 96,623 km^2^ of Africa’s largest inland basin, the Lake Chad Basin (*54*). Lake Chad is an endorheic freshwater lake primarily fed by the southeastern Chari-Logone River, where annual maximum and minimum runoff occur in December and April, respectively (*13*, *55*). The variable inflow of the river drives marked fluctuations in the water depth of the shallow lake, submerging the flat floodplains extensively (*56*). Different inundation frequencies result in diverse land cover, including open water, marshes, swamps, savanna grassland, and sparse shrublands. In this study, we define total inundated areas as the sum of open water and extensive inundated vegetation.

The climate of the Lake Chad region is tropical arid with a wet season from May to September and a dry season from November to April. Annual precipitation averages 560 mm at the southern border of Lake Chad and about 250 mm at the northern border. Seasonal temperatures vary substantially, ranging from 27° to 35°C in the wet season and 20° to 27°C in the dry season. The shifts in the seasonal location of the Intertropical Convergence Zone usually lead to drought and floods that directly influence the wetland water area (*57*). Since 2019, the Lake Chad region has experienced a wetter climate and recovered its earlier water extent. Extreme flooding in the 2020 rainy season, marked by record precipitation, led to the rapid increase of wetland inundation areas (*58*, *59*).

CH_4_ emission enhancements over the Lake Chad region before 2019 were previously estimated using process-based wetland emission models and satellite-based model inversion (*60*, *61*). However, large discrepancies persist in the magnitude of emissions on monthly and annual timescales, suggesting uncertainties that may stem partly from the prior model information used in the inversions, or from poorly understood factors controlling wetland dynamics in this region. For example, the rapid recovery of water areas in recent years and its potential impact on Lake Chad CH_4_ emissions require further investigation.

### TROPOMI methane observations

TROPOMI was launched in October 2017 onboard the polar-orbiting Sentinel-5 Precursor satellite, which provides daily methane observations at ∼13:30 local overpass time with a swath width of 2600 km and a nadir ground pixel size of 5.5 km by 7 km (7 km by 7 km before 6 August 2019) (*62*, *63*). In this study, we used the TROPOMI WFM-DOAS (TROPOMI/WFMD v1.8) daily column-averaged methane dry-air mole fractions (XCH_4_) product (*64*) to calculate monthly CH_4_ emissions. WFMD v1.8 is a scientific methane product that simultaneously retrieves the atmospheric column-averaged dry-air mole fractions of methane (XCH_4_) and carbon monoxide (XCO) in the 2.3-μm spectral range of the short-wave infrared (SWIR). WFMD data are also corrected for any known retrieval biases such as destripping, surface-albedo dependence, and topography effects (*64*, *65*). Only high-quality XCH_4_ under clear-sky scenes with minor scattering by aerosols and optically thin clouds (i.e., cirrus) is released. To exclude potential uncertainties due to individual scattering or shielding events at desert dust or cloud edges, we also filter pixels with high aerosol optical depth (AOD). Specifically, our baseline calculations exclude pixels with AOD values exceeding 0.75 at 550 nm, following the methodology of Liu *et al.* (*66*). This threshold balances the impact of aerosol interference with the number of valid samplings on emission estimates.

Uncertainties related to aerosols are more complex than simply filtering by AOD. They are influenced by specific aerosol scenarios in different locations, size distributions, types, and other factors. While a detailed discussion of each aspect falls beyond the scope of this paper, we conducted a sensitivity study using all pixels to evaluate their uncertainty in our study area (see the next section and table S3).

We performed the filtering using daily Moderate Resolution Imaging Spectroradiometer (MODIS/Aqua) C6.1 AOD observations (*67*), which aligns closely with TROPOMI overpass times. Integrating these with the Copernicus Atmosphere Monitoring Service reanalysis dataset ensures that all pixels have an available estimated AOD (*66*).

### Divergence method and uncertainty

The innovative divergence method is based on the mass balance theory and stands out for both its speed and no need for a priori knowledge of the spatial distribution of methane sources. We use the divergence method to derive monthly CH_4_ emissions from TROPOMI at a horizontal resolution of 0.2° over Lake Chad, providing rare insights in wetland emissions. The divergence method was initially developed for NO*_X_* emissions estimates (*68*) and later also applied for other species such as sulfur dioxide (SO_2_) (*69*) and methane (*53*, *66*, *70*). The divergence method has been widely applied in methane emission estimates in recent studies and rigorously validated using model simulations (e.g., GEOS-Chem and WRF-Chem) and bottom-up emission inventories (*66*, *70*). The preliminary CH_4_ emissions, $E^{\prime }$, over a certain period is described by

$$E^{\prime }=\bar{D_{d}^{S}}=\bar{\nabla \left[\left(X_{d}^{L}-X_{d}^{B}\right)\times A_{d}^{L}\;\vec{w}\right]}$$ (1)

where $D_{d}^{S}$ is the daily divergence of a source. $X_{d}^{L}$ is the daily XCH_4_ within 500 m near the ground, which is estimated by subtracting the vertical column of methane above 500 m from the TROPOMI total column observations. Estimating the XCH_4_ in the lower atmosphere is crucial as enhancements due to the transport in the upper atmosphere cannot be directly linked to the ground emissions. The vertical column above 500 m that we use is the fourth generation European Center for Medium-Range Weather Forecasts (ECMWF) Atmospheric Composition Reanalysis (EAC4) dataset at a relatively high spatial resolution with daily frequency, 0.75° horizontally and 60 layers vertically (*71*), with methane serving as a background species for chemical reactions. The spatial distribution of CH_4_ in the EAC4 dataset is mainly driven by transport and orography, because it contains no CH_4_ emissions in its simulation, while using a global ground-based measurements network to constrain methane concentrations at the background level near the surface. Specifically for this, the National Oceanic and Atmospheric Administration Global Monitoring Laboratory flask observations of CH_4_ at background locations for the years 2003–2014 were used to derive a zonally varying seasonal cycle and decadal trend at the surface (*72*).

It is important to note that the total dry air column from the EAC4 dataset is constrained by the TROPOMI retrieval for each pixel, which guarantees mass conservation. $X_{d}^{B}$ is the regional background of $X_{d}^{L}$, which is defined as the average of the lower 10th percentile of its surrounding ±3 grid cells in the zonal direction and meridional direction (7 × 7 = 49 grid cells in total by taking the current grid cell as the center). $X_{d}^{B}$ is built if more than 10 grid cells have valid retrievals in this domain. $A_{d}^{L}$ is the corresponding air density column below 500 m. The daily wind field is obtained halfway the height of the Planetary Boundary Layer, close to the overpass time from the European Centre for Medium Range Weather Forecasts. Wind speeds are constrained to less than 10 m/s because extremely high wind speeds lead to lower regional gradients and, therefore, more noise on the emission estimates. The divergence of the daily wind field has been iteratively removed to produce a daily nondivergent wind field ($\vec{w}$), following the method described in Bryan (*73*). Using nondivergent wind fields can largely reduce the spatial feature of local wind-field changes induced by complicated orography, which would, otherwise, propagate into the $D_{d}^{S}$ calculation (*66*, *74*).

The divergence method for estimating emissions is applicable over longer periods but can be quite noisy for individual days. Thus, we filter out grid cells with fewer than 5 valid observation days for monthly emissions calculations or fewer than 10 days for yearly emissions estimates. In addition, the inhomogeneous spatial distribution of the divergence of the background ($\bar{D_{d}^{B}}=\bar{\nabla \left(X_{d}^{B}\times A_{d}^{L}\right)\;\vec{w}}$) indicates the possible residue of the regional background we built in Eq. 1. Therefore, we evaluate the contribution from the residue background for each grid cell with positive $E^{\prime }$ by checking the spatial correlation between $\bar{D_{d}^{B}}$ and $\bar{D_{d}^{S}}$ in the domain. The contribution from the remaining background is then subtracted from the preliminary estimated emissions, $E^{\prime }$, to derive the final emission estimates. The detailed procedure can be found in the Supplementary Materials.

In Fig. 1A, we report an uncertainty range of emissions, which mainly represents the contribution from the three aspects discussed below: (i) pixels with high AOD values, which has been mentioned in the “TROPOMI methane observations” section; (ii) missing data due to cloud cover or retrieval failures; and (iii) regional background that we built for the divergence calculation. Table S3 summarizes the settings of our baseline and three sensitivity tests to evaluate the uncertainties of these on the final emission estimation.

We found that pixels with AOD of >0.75 typically occur far away from the Lake Chad region, near the borders of our study domain (where one finds the transition from grasslands to deserts). The impact of aerosols on annual XCH_4_ and emissions varies by location (see results of 2020 presented in fig. S7 as an example). The uncertainties due to aerosols are smaller than for the other two uncertainty aspects. The relative uncertainty of monthly total methane emissions due to aerosols ranges from −20 to 30%, suggesting a relatively even/random distribution of the uncertainty.

The most common months with missing observations are June to August, primarily due to cloud cover (figs. S8 and S9). To evaluate the impact of missing data on the final emission estimates, we first count the number of grid cells with detected sources for the entire year ($N_{y}$) and for each month in that year ($N_{y,m}$). To estimate the potential underestimation due to missing data, we randomly select $N_{y,m}$ grid cells from the yearly emission field and calculate the yearly total emissions based on these selected cells. The ratio ($R_{m}$) of the total emissions from $N_{y,m}$ grid cells to the total emissions from $N_{y}$ grid cells indicates the possible percentage of underestimation caused by missing data. If more than 50% of the grid cells with detected sources are missing in a given month, we repeat the random selection process 30 times and calculate the yearly total emissions for each subset. Otherwise, we repeat the process 10 times to get subsets, {$R_{m}$}. The uncertainty due to missing data in a certain month is then estimated by

$$\delta =E_{y,m}\left[\frac{1}{\text{Med}\left(\{R_{m}\}\right)}-1.0\right]$$ (2)

$E_{y,m}$ stands for the emission of a month (m) estimated by the divergence method, and $\text{Med}\left(\{R_{m}\}\right)$ is the median value of subsets, {$R_{m}$}. The smallest value of $R_{m}$ occurs in August for each year, when only about 20% of the sources are captured by TROPOMI observations. The influence of missing data can result in an underestimation of monthly methane emissions by up to 50%, which can be considered the upper bound of the final emission estimates. This issue is particularly pronounced during months with high data gaps, such as June to August, when cloud cover reduces the availability of satellite observations.

The last aspect is the regional background that we built. In the case of our baseline, the regional background for each grid cell is defined as the mean of the lower 10th percentile of its surrounding 7 by 7 grid cells. Here, we tested a very high background by using the median value of its surrounding 7 by 7 grid cells. This high regional background minimizes the selection of low observations as the background, which can be considered the lower bound of our estimated emissions. As a result, the estimated emissions are notably lower in most locations (fig. S10). Sensitivity tests revealed that the choice of regional background can introduce the highest uncertainty, ranging from −2.4 to 0.3 Tg year^−1^, and can lead to an overestimation of up to 50% in our monthly baseline estimates.

### Anthropogenic and wetland emission inventories

In this study, we compared our derived CH_4_ emissions with other anthropogenic and natural CH_4_ emission inventories. We evaluated potential anthropogenic methane sources in the Lake Chad region using the Emissions Database for Global Atmospheric Research (EDGAR) v8.0, the current version of this widely used inventory. It provides sector-specific annual emission maps from 1970 to 2022 on a grid of 0.1° (*75*). Following the approach of Liu *et al.* (*66*), we categorized CH_4_ source sectors from EDGAR into three groups: agriculture, waste, and energy (fig. S11). Compared to our derived emissions, the contribution of anthropogenic emissions inferred by EDGAR is only about 15%, indicating that natural sources dominate in this region.

Wetland CH_4_ emissions estimated by process-based models are less frequently updated than anthropogenic emission inventories. In this study, we compared our results with three wetland emission datasets: WetCHARTs v1.3.3 for 2019 (*29*), ORCHIDEE-MICT, and the LPJ-wsl model simulations from Peng *et al.* (*6*) for 2019 to 2020. WetCHARTs provides monthly global wetland emissions using an ensemble of 18 model configurations from 2001 to 2019. These models encompass primary uncertainties by using multiple global scale factors, heterotrophic respiration models, CH_4_:C temperature dependencies, and four wetland extent scenarios [Global Lakes and Wetlands Database or Global Land Cover wetland extent calibrated by ERA5 or Surface Water Microwave Product Series (SWAMPS)] (*29*). The ORCHIDEE-MICT model used eight driver scenarios comprising four climate forcings and two wetland extent datasets on a 1° grid. The two sets of wetland area dynamics were simulated by the topography-based hydrological model (TOPMODEL) and calibrated by two static wetland maps: Regularly Flooded Wetlands static map and Global Inundation Estimate from Multiple Satellites version 2, respectively. For LPJ-wsl, they used the same four climate forcing datasets and TOPMODEL-based wetland dynamics calibrated using a combination of SWAMPS and GLWD (*76*).

### Open and vegetated water mapping and validation

We processed 7756 images Sentinel-2 imagery covering the study area between January 2019 and December 2022 on Google Earth Engine. This analysis used Sentinel-2 Level-1C surface reflectance (SR) imagery, including atmospheric and geometric corrections. The Sentinel-2 satellite carries a multispectral instrument (MSI) that captures bands from visual to SWIR with 10- or 20-m spatial resolution and 5- to 10-day return interval. We removed images with cloud probabilities greater than 50% and mosaicked same-date images into a single composite to eliminate double-counting along overlapping image edges (*28*).

We extracted monthly open water and inundated vegetation areas using the prior knowledge-based algorithm and spectral indices (Eqs. 3 to 5) from Sentinel-2 imagery. The three main steps involved the following (fig. S12): (i) Because pure water and plant-water interactions produce unique signals, we identified water when land surface water index (LSWI) values temporarily exceeded normalized difference vegetation index or EVI (*77*, *78*). We used a water signal frequency [Num. (*Y*_w_)] greater than 1 per year to distinguish water from no-water surfaces. Based on the yearly mean EVI (EVI_yearlymean_). We further classified water into yearly open water (EVI_yearlymean_ < 0.1) and inundated vegetation (EVI_yearlymean_ ≥ 0.1). (ii) Monthly open water was identified when the time series of yearly open and inundated vegetation pixels showed a monthly mean EVI value (EVI_monthlymean_) below 0.1 and a monthly water signal frequency [Num. (*Y*_w_)] above 1. (iii) To mitigate underestimating wetland inundation areas due to dense vegetation, we used LSWI time series before and during the vegetation growth phase to identify water during the closed canopy phase of yearly inundated vegetation.

Here, we determined the peak of the growing season (POS) following Yang *et al.* (*79*), identifying the last water signal before POS (P_1_), the first LSWI < 0 before POS (P_0_), and the first water signal or LSWI < 0 after POS (P_2_). The period from P_0_ to P_2_ was identified as nonwater phase if the LSWI time series from P_1_ to POS remained below 0 (*Y*_P1-POS_). The period from P_1_ to P_2_, excluding the nonwater phase, represents inundated vegetation. During 2019 to 2021, more than two valid observations existed each month in our study areas for producing monthly cloud-free maps (fig. S9).

We validated monthly surface water using PlanetScope imagery [5-m resolution; sensor: Dove Classic (PS2), Dove-R (PS2.SD), and SuperDove (PSB.SD); www.planet.com/basemaps/]. The PlanetScope constellation provides nearly daily global coverage with each scene ranging from ∼280 to 630 km^2^ in size and four bands (red, green, blue, and near-infrared) (*80*). We selected ∼3420 high-resolution images per month (a total of 13,680 images) across the region. Validation points were randomly generated across each monthly composite image (total of 3264 points) and were visually interpreted as nonwater, open water, or inundated vegetation. We calculated the confusion matrix, including omission error, commission error, and overall accuracy between the nonwater class and both open water and inundated vegetation class and between open water and inundated vegetation to assess classification performance (*28*)

$$\text{LSWI}=\frac{\rho_{\text{nir}}-\rho_{\text{swir}2}}{\rho_{\text{nir}}+\rho_{\text{swir}2}}$$ (3)

$$\text{NDVI}=\frac{\rho_{\text{nir}}-\rho_{\text{red}}}{\rho_{\text{nir}}+\rho_{\text{red}}}$$ (4)

$$\text{EVI}=2.5\times \frac{\rho_{\text{nir}}-\rho_{\text{red}}}{\rho_{\text{nir}}+6.0\rho_{\text{red}}-7.5\rho_{\text{blue}}+1}$$ (5)

where $\rho_{\text{blue}}$, $\rho_{\text{red}}$, $\rho_{\text{nir}}$, and $\rho_{\text{swir}2}$ are SR values for the blue band [496.6 nm (S2A)/492.1 nm (S2B)], red band [664.5 nm (S2A)/665 nm (S2B)], near infrared band [835.1 nm (S2A)/833 nm (S2B)], and short wave infrared red 2 band [2202.4 nm (S2A)/2185.7 nm (S2B)] in the Sentinel-2 MSI sensor.

We applied auxiliary wetland/water datasets to evaluate interannual and monthly water area variations. The Database for Hydrological Time Series of Inland Waters (DAHITI) reconstructed water level time series using a combination of altimeter missions, such as Sentinel-3A, Sentinel-6A, and Jason-2 (*81*). We used monthly mean values derived from observations with 10- to 25-day temporal resolutions. The Jet Propulsion Laboratory RL06.1_v03 mascons data from Gravity Recovery and Climate Experiment Follow-on mission provides monthly estimates of terrestrial water storage changes, including soil moisture, groundwater, surface water, and wetlands (*82*). The Wetland Area and Dynamics for Methane Modeling (v2.0) was generated at a monthly time step for 2000–2020 based on SWAMPS v3.2 and multidataset fusion, excluding ocean, lakes, ponds, rivers, streams, and irrigated rice paddies (*83*). The Global Wetland Dynamics Dataset (v4.0) for the period 1980–2020 at a 0.25° spatial resolution was produced using TOPMODEL, calibrated with a combination of four observation-based wetland datasets and seven gridded soil moisture reanalysis datasets (*20*). The Joint Research Center global monthly surface water dataset (30-m resolution, 1984–2021), which excludes inundated vegetation, was generated from high-resolution Landsat-5, Landsat-7, and Landsat-8 imagery (*84*). Another product provides monthly binary surface water extent maps at a 0.01° by 0.01° resolution using the UC Berkeley Random Walk Algorithm WaterMask applied to CYGNSS (*85*). The onboard L-band receiver of CYGNSS has the potential to detect surface water beneath dense canopies.

### Seasonal CH_4_ emission hysteresis

We apply a quadratic equation to calculate the emergent dependence of CH_4_ emissions on wetland inundation areas during expansion and reduction phases of the wetland dynamics. If the wetland inundation area in a given month is larger than in the previous month, then it is classified as the expansion phase; otherwise, it is considered the reduction phase. Two metrics are used to quantify the observed seasonal CH_4_ hysteresis: (i) The normalized area of seasonal CH_4_ hysteresis ($H_{A}$), defined as the area enclosed by emergent dependencies of CH_4_ on wetland inundation area inferred from expansion (earlier) and reduction (later) stages of wetland inundation dynamics (Eq. 6); and (ii) the mean seasonal CH_4_ hysteresis (*H*_µ_), defined as the difference between mean monthly CH_4_ emissions inferred from measurements taken between reduction and expansion stages of the wetland inundation area. These two metrics are conceptually similar to those used to quantify temperature hysteresis in wetland CH_4_ emissions (*25*). We excluded monthly data if, for a given month, the number of valid TROPOMI observations is less than two or if more than 70% of the region lacks valid observations (fig. S9). The results without data exclusion are provided in fig. S13

$$H_{A}=\frac{\int_{A_{\text{min}}}^{A_{\text{max}}}\left[E_{{\text{CH}}_{4},\;\text{later}}\left(A\right)-E_{{\text{CH}}_{4},\;\text{earlier}}\left(A\right)\right]\mathrm{dA}}{\text{max}\left\{\text{abs}\left[E_{{\text{CH}}_{4},\;\text{earlier}}\left(A\right),E_{{\text{CH}}_{4},\;\text{later}}(A)\right]\right\}\;\cdot \left(A_{\text{max}}-A_{\text{min}}\right)}$$ (6)

## Supplementary Materials

### This PDF file includes:

Supplementary Texts 1 to 5 Figs. S1 to S15 Tables S1 to S4 References
