# Supplementary material for: Unveiling cascading lag effects of wetland methane emissions: Evidence from Lake Chad in Africa
Source: Sci Adv. 2026 Jul 29;12(31):eadx9866. doi: 10.1126/sciadv.adx9866 (PMC13418730; doi:10.1126/sciadv.adx9866)
Supplement: Supplementary file 1 — Supplementary Texts 1 to 5 Figs. S1 to S15 Tables S1 to S4 References [file sciadv.adx9866_sm.pdf]

Science Advances
AAAS

Supplementary Materials for

Unveiling cascading lag effects of wetland methane emissions: Evidence from Lake Chad in Africa

Ruoqi Liu *et al.*

Corresponding author: Geli Zhang, geli.zhang@cau.edu.cn; Mengyao Liu, mengyao.liu@knmi.nl

Sci. Adv. **12**, eadx9866 (2026)
DOI: 10.1126/sciadv.adx9866

This PDF file includes:

Supplementary Texts 1 to 5
Figs. S1 to S15
Tables S1 to S4
References

Supplementary Text 1. Deriving the final CH_4 emissions

The advantages of including X_d^B in Eq. (1) are (i) it can be used to diagnose the contribution of inhomogeneous background, especially over mountains and coastal regions, and (ii) the system biases between CAMS and TROPOMI, which leads to biased X_d^L, are included in both and can be greatly reduced by subtracting X_d^B from X_d^L. The inhomogeneous spatial distribution of the divergence of the background ($\overline{D_d^B} = \overline{\nabla(X_d^B \times A_d^L) \vec{w}}$) indicates the possible residue of the regional background we built in Eq. (1). Therefore, we evaluate the contribution from the residue background for each grid cell with positive E' by checking the spatial correlation between $\overline{D_d^B}$ and $\overline{D_d^S}$ (the divergence of the source) in the domain. For grid cells with positive E', a linear regression is applied to its surrounding ±3 cells:

$$y_i = k \cdot x_i + b \qquad \text{(S1)}$$

where y_i stands for $\overline{D_d^S}$ and x_i stands for $\overline{D_d^B}$ of grid i. k and b are the slope and intercept of the linear regression, respectively. If Eq. (S1) is applicable to the center grid, it implies the residue of the background still contributes to E' and should be subtracted. This linear correlation can be distinctive over locations with large variations in orography (e.g., mountains, coastal areas). If more than 68% of the grid cells and the grid cell itself fall within the prediction lines of Eq. (S1), estimated emissions are set to zero because $\overline{D_d^S}$ can be fully predicted by $\overline{D_d^B}$ according to Eq. (S1). The grid cells are considered to be influenced by residue background only when Eq. (S1) is significant (p-value < 0.01), and they are further corrected by the spatial correction:

$$E^{corr} = E' - (k \cdot \overline{D_d^B} + b) \qquad \text{(S2)}$$

in which $(k \cdot \overline{D_d^B} + b)$ is regarded as the contribution from the remaining background, which should be subtracted from the preliminary estimated emissions, E'. The orography of Lake Chad is relatively smooth, meaning that Eq. (S1) is significant to very few grid cells. In addition, we further filter emissions by setting the values of grid cells with E^{corr} < 1kg/km^2/h to zero, as these sources are too low to be distinguished from the regional background level. More details about the corrected emissions and their evaluation can be referred to Liu *et al* (*66*).

Supplementary Text 2. Isolation of the non-wetland methane emissions

To better estimate the contribution from wetland emissions, we need to isolate the impacts of other anthropogenic emissions in the region. In Fig. 1, we used the EDGAR v8 emission inventory to provide us with a bottom-up estimate of the, assumingly relatively small, anthropogenic methane sources in the Lake Chad region with a total of 0.45 Tg a^{-1} (0.2-0.5 Tg a^{-1}, uncertainties among the minimum tropomi-based monthly total CH_4 emissions). Subtracting these non-wetland sources (0.45 Tg a^{-1}) from the observed 2019–2022 annual mean total emissions (3.43 Tg a^{-1}), we find 2.98 Tg a^{-1} as our estimate for the 2019–2022 annual mean wetland CH_4 emissions in the Lake Chad region.

As much available information as possible from various sources was collected, including bottom-up emissions inventories, catalogs of large methane emitters based on satellite observations, and locations of oil/gas plants. Anthropogenic emissions generally originate from two types of sources: isolated sources, such as oil/gas production facilities and landfills, and area-like sources, such as rice paddies or livestock. For isolated sources, we found no reported emissions in catalogs based on target satellite data (e.g., EMIT (*87*)), which are typically used to pinpoint facilities, nor oil/gas plant database (e.g., Global Energy Monitor (*88*)). Regarding area-like sources, one contributor is rice paddy growth, but Supplementary Fig. S14 indicates no rice paddy cultivation in the area. Another important source is livestock, primarily associated with enteric fermentation and manure management. In Sub-Saharan Africa, emissions from animals can exhibit seasonal variations due to changes in feed quality, quantity, and intake (*89, 90*). However, spatial

distributions of livestock can also vary with time. For example, rangeland fodder resources, which are used to feed ruminants, decline considerably in both quantity and quality in the Lake Chad region from the wet to dry seasons, so Yaéré floodplain pastures (Chari-Logone River Basin, near the southern border of Chad) become an important habitat for ruminants during dry seasons (typically November to March) (*91*). We didn't find a strong seasonality of methane emissions from livestock based on the latest version (v8) of EDGAR, where the activity data for the agricultural sectors originate primarily from FAO (2022) (https://www.fao.org/faostat). Meanwhile, Fig. 2E also shows much lower methane emissions from livestock than our derived emissions. Therefore, the strong seasonality observed over the Lake Chad region is unlikely to be caused by livestock.

Supplementary Text 3. Satellite-derived water areas and their seasonality

We used remote sensing data and a phenology-based algorithm to delineate monthly water areas at 10 m resolution from 2019 to 2022. The resulting monthly maps of open water and inundated vegetation all had high overall accuracies (about 90%) (Table S4). The area of the total water and inundated vegetation showed a substantial annual variability, with the largest areas in 2020 (20245 km^2 and 14423 km^2, respectively), while the open water areas were the smallest (3677 km^2) in 2019, and gradually increased to 5852 km^2 in 2022. The annual variations in water areas were mainly attributed to the changes in open water in the northern pools and inundated vegetation in the southern floodplains (Supplementary Fig. S15). The spike in total water areas could be partly explained by the floods in this region in the summer and autumn of 2020 and 2022 (*59*). The monthly changes in open water areas were consistent with the JRC surface water areas (Supplementary Fig. S2a). The inundated vegetation extent variations were consistent with TOPMODEL-based wetland dynamics in 2019 and 2020, but were opposite to the SWAMPS-based wetland extent (WAD2M) (Supplementary Fig. S2b). The seasonal cycle of our total water area variations was similar to the inundation extent of Berkeley-RWAWC (Supplementary Fig. S2c), and the changes in the water level of Lake Chad, indicated by the site measurements (Fig. 1C).

Supplementary Text 4. Magnitude of CH_4 emissions in Lake Chad

The CH_4 emissions over the Lake Chad region based on TROPOMI observations are significant with high confidence, and are on average 3.43 Tg a^{-1} during the period 2019–2022 (Fig. 1B). This estimate is much larger than indicated by bottom-up non-wetlands anthropogenic emission inventories of 0.45 Tg a^{-1} (0.2-0.5 Tg a^{-1}, uncertainties among the minimum TROPOMI-based monthly total CH_4 emissions) according to EDGAR v8 emission inventory and other anthropogenic sources information (see the Supplementary Materials for more details), suggesting that natural wetlands are the dominant contributor to CH_4 emissions in this region. We find 2.98 Tg a^{-1} as our estimate for the 2019–2022 annual mean wetland CH_4 emissions in the Lake Chad region. We identified a dramatic increase in wetland CH_4 emissions for the Lake Chad region from 2019 to 2020 (0.80 Tg a^{-1}) due to extremely high precipitation and inundation area in 2020, which contributes to 3–12 % of increases in global CH_4 emissions and 16 ± 3.7% of increases in global wetland CH_4 emissions (*6*). This indicates that the Lake Chad region is likely a substantial contributor to continental/global wetland-related emissions.

Compared to earlier studies based on satellite observations (Fig. 1E), our estimates of methane emissions of the whole region (on average 3.43 Tg a^{-1}, 2019–2022) agree with Lunt's study for 2010–2016 (prior: ~2.00 Tg a^{-1}; posterior: ~4.31 Tg a^{-1}) based on GOSAT observations and GEOS-Chem inversion techniques (*60*). Recently, strong enhancements were also detected by TROPOMI. Vanselow *et al* (*92*) detected continuous sources (on average 1.39 ± 0.3 Tg a^{-1}, 2018–2021) over the southern Lake Chad region based on the records during 2018-2021 by using the

persistent hotspot detection (PHD) algorithm. The European Space Agency methane monitoring platform also labeled the Lake Chad region as a sample with a higher concentration of methane plumes in 2019. These studies identified this region as an unknown source, but we further determined the distribution, source, and mechanism of emissions from Lake Chad that have not been previously documented. However, the comparison with McNorton's emission estimate in the same area (southern pools of Lake Chad) in 2019–2020 showed that our estimate (0.90 Tg a^{-1}) was higher than that of McNorton's (prior: 0.35 Tg a^{-1}; posterior: 0.38 Tg a^{-1}) (*61*). The differences and uncertainties in the derived emissions can predominantly be attributed to the limited sampling and the use of different methodologies for emission inversion. For example, only 65 out of 485 days of operational TROPOMI observations were available during McNorton's study period (*61*). Also, the spatial coverage of GOSAT observations (~15 days for a global image) used by Lunt (*60*) is even sparser than TROPOMI. Furthermore, the atmospheric model inversions in their studies rely on bottom-up emission inventories and precipitation patterns to adjust prior emissions, which are less favorable for identifying unknown sources. In contrast, our emission estimates are derived from satellite observations only and are independent of prior assumptions about CH_4 emissions in the region.

Our derived wetland emissions scale to about 13% of the top-down derived natural emission estimate by Saunois *et al* (*93*) for Equatorial Africa. Compared to the wetland methane emissions from this study, the bottom-up wetland emission estimates, such as WetCHARTs (*29*), LPJ-wsl (*12*), and ORCHIDEE-MICT (*6*) over the Lake Chad region are much lower (Fig. 1 D, E), although open water is not included in their simulations as a minority (diffusive CH_4 from Lake Chad was about 0.09 Tg a^{-1} (*30*)). The Lake Chad region has an extremely fragmented landscape with dry land, seasonally and permanently inundated wetlands. The wetland dynamics are easily missed by these coarser bottom-up wetland emission estimate models that use wetland area data in grid cells of 0.5° or larger (Fig. 1D). We argue that small-sized tropical wetlands (less than 0.1 km^2) can still contribute a relatively large fraction of the total wetland CH_4 emissions (*32*). High-resolution observation-based wetland maps enable us to capture the impacts of variations among- and within-wetlands on CH_4 emissions, which is usually characterized by a spatially heterogeneous and temporally intermittent nature (*33*).

Supplementary Text 5. Improvements of the ORCHIDEE model

We took the ORCHIDEE model as an example and conducted recalibrated simulations by incorporating the identified cascading lag mechanism and our monthly wetland areas. Our results demonstrate that these new mechanistic constraints substantially improve model performance in simulating tropical wetland CH_4 estimates, achieving strong alignment in satellite observations (RMSE = 0.14) in both seasonal magnitude and phase (Supplementary Fig. S6).
The specific model modifications are is as follows (the baseline version is named ORCHIDEE_baseline, and the version with two new mechanisms is named ORCHIDEE_M1+M2):
Mechanism I: Substrate Accumulation & Lag. We modified the substrate module to account for seasonal fluctuation of litter inputs. During the expansion phase (wet season, June–October), submerged vegetation contributes to substrate accumulation within the anaerobic soil environment. The subsequent decomposition and CH_4 production create a natural temporal lag between the peak of flooding and the peak of emissions in the ORCHIDEE_M1+M2.
Mechanism II: Dynamic Vascular Transport. We refined T_veg, a parameter in the model representing the transport capacity of plant aerenchyma. In the baseline version of ORCHIDEE, T_veg is a constant. We implemented a seasonal T_veg to reflect the vegetation succession. Specifically, since vegetation is submerged and partially inhibited as the inundation area expands, and then re-emerges as the inundation area recedes, T_veg is set to 0.5/3 during the inundation

expansion phase (June–October) and is linearly increased to 0.5 during the receding phase (November–next May).
This seasonal variation in T_veg effectively captures the enhanced CH_4 transport from the soil to the atmosphere via the developed vascular systems of newly exposed plants. The comparison between TROPOMI inversion, ORCHIDEE_baseline, and ORCHIDEE_M1+M2 is shown in Supplementary Fig. S6.

Fig. S1 Spatial distributions of wetland for the study domain.

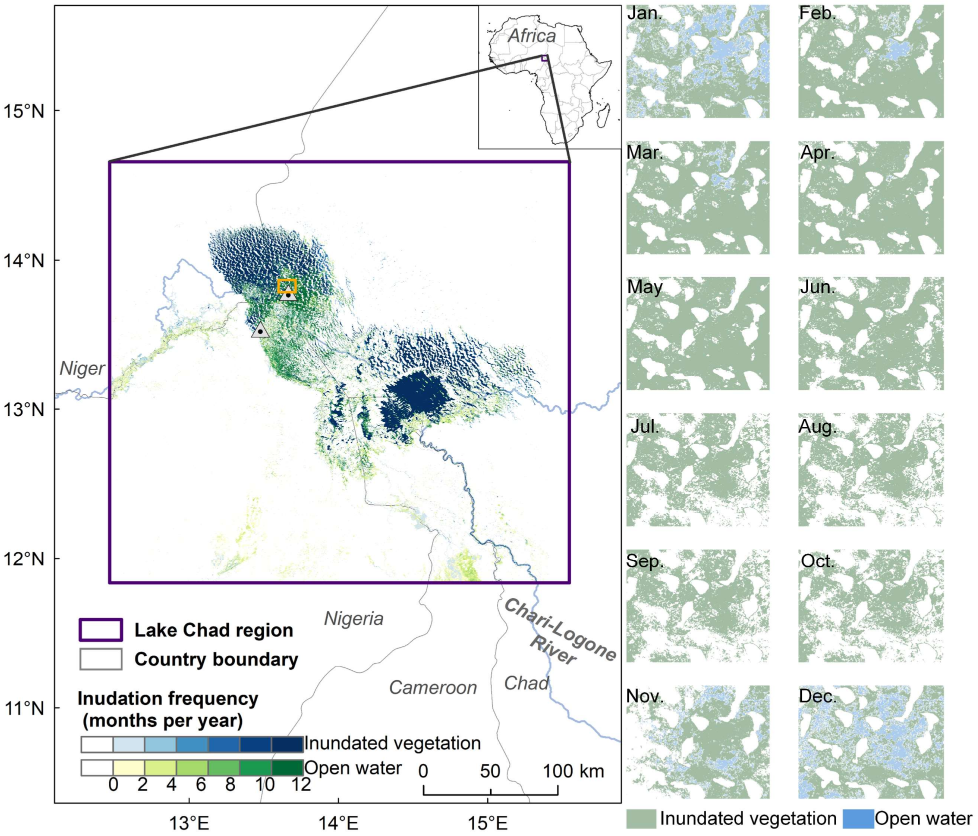

The left panel is the frequency of inundated vegetation and open water (months per year) in the study domain (indicated by a purple rectangle). The right panels provide a zoomed-in view of wetland dynamic areas.

Fig. S2 Seasonal dynamics of retrieved monthly wetland areas and their validation for the Lake Chad region.

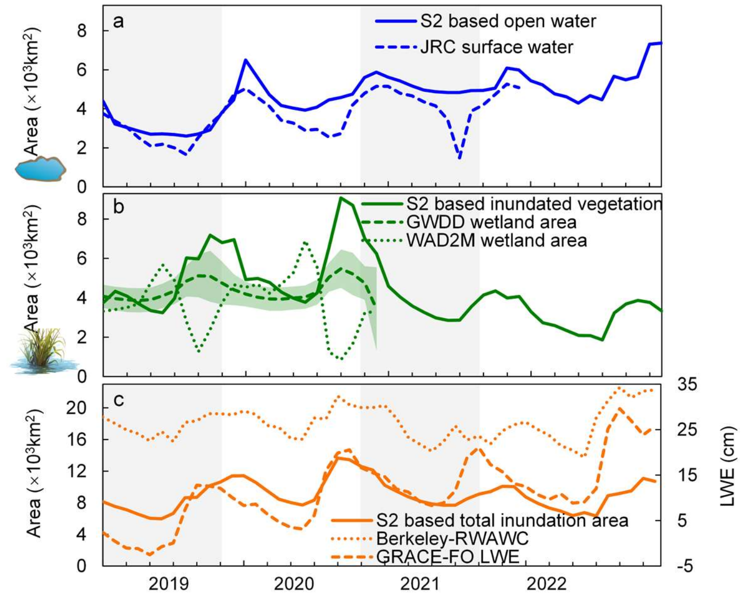

Monthly derived (a) open water areas, (b) inundated vegetation areas, and (c) total inundation areas from Sentinel-2 for the years 2019 to 2022. These results are compared with surface water area from JRC, wetland area from GWDD and WAD2M reprocessing models, and total water area from Berkeley-RWAWC and GRACE-FO Liquid Water Equivalent (LWE).

Fig. S3 Time lag between CH_4 emissions, water areas, and environmental factors.

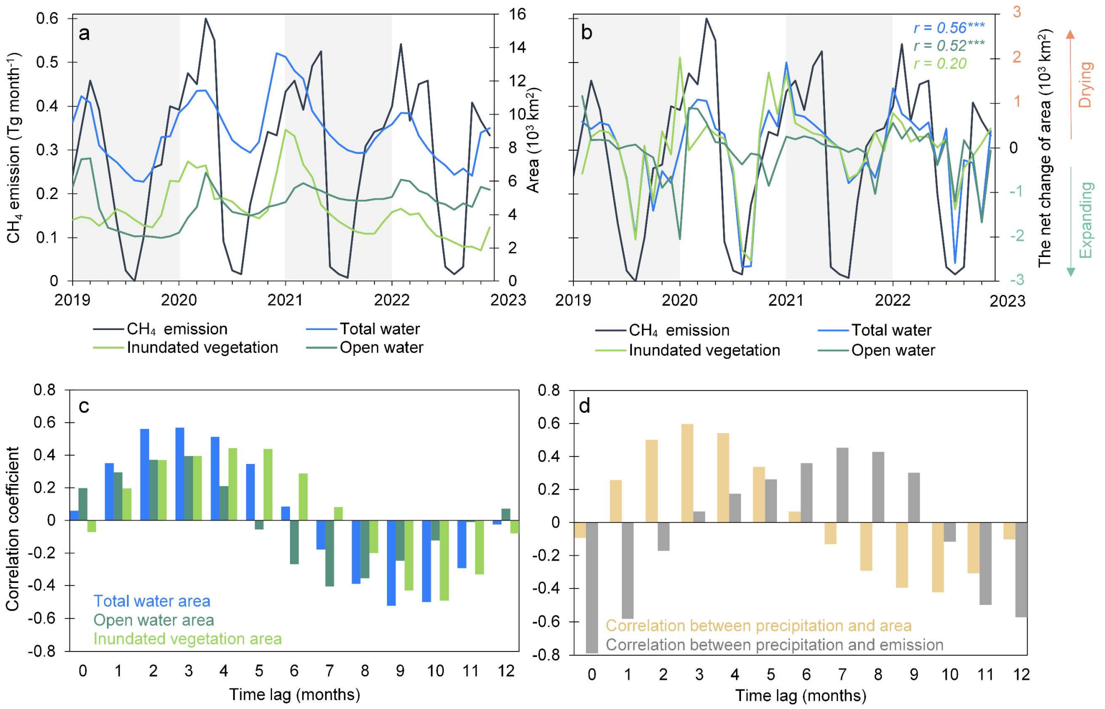

(a) Time series of monthly CH_4 emissions (Tg month^{-1}) and water areas (10^3 km^2) during 2019–2022. (b) Time series of monthly CH_4 emissions and net change of water areas (10^3 km^2). Note that the positive net changes of water areas (opposite of (certain month – former month)) mean that water areas contracted in a certain month. (c) Correlation coefficients between CH_4 emissions and water areas with different time lag choices. (d) Correlations between monthly precipitation and monthly total water area, and monthly CH_4 emissions with different time lag choices. Precipitation is collected from the Global Precipitation Measurement (GPM) IMERG Final version 07. The cross-correlation results based on other precipitation products are presented in Table S1, S2.

Fig. S4 Spatial distributions of characteristics of the inundation in the Lake Chad region.

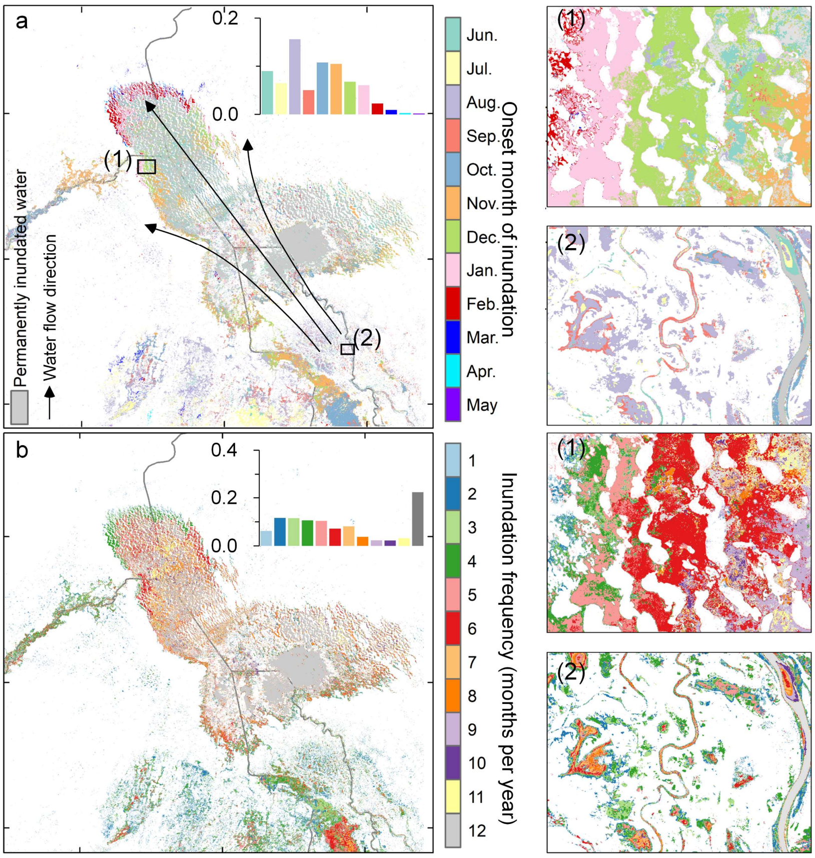

(a) Onset month of inundation since the month with the minimum water area. (b) Inundation frequency (months per year). The time period is from June 2019 to May 2020.

Fig. S5 Time series curves of two submerged sample points based on Sentinel-2 images.

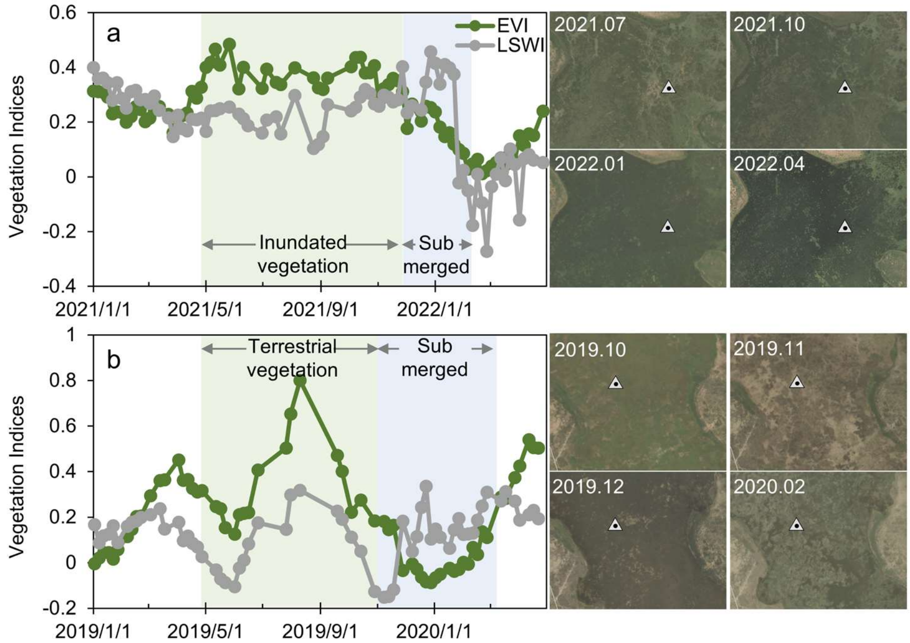

(a) inundated (13.66556°E, 13.77940°N) and (b) terrestrial vegetation (13.47812°E, 13.53406°N). The locations of these two points are indicated in Figure 1. LSWI greater than or equal to EVI and reduced EVI indicates that vegetation is submerged due to inundation (*77, 78*). The RGB images shown on the right were generated from Sentinel-2 data. Sentinel-2 data are provided by Copernicus/European Space Agency (ESA) (*94*).

Fig. S6 The comparison between TROPOMI inversion, the original ORCHIDEE version (ORCHIDEE_baseline), and the version with the two mechanisms implemented (ORCHIDEE_M1+M2).

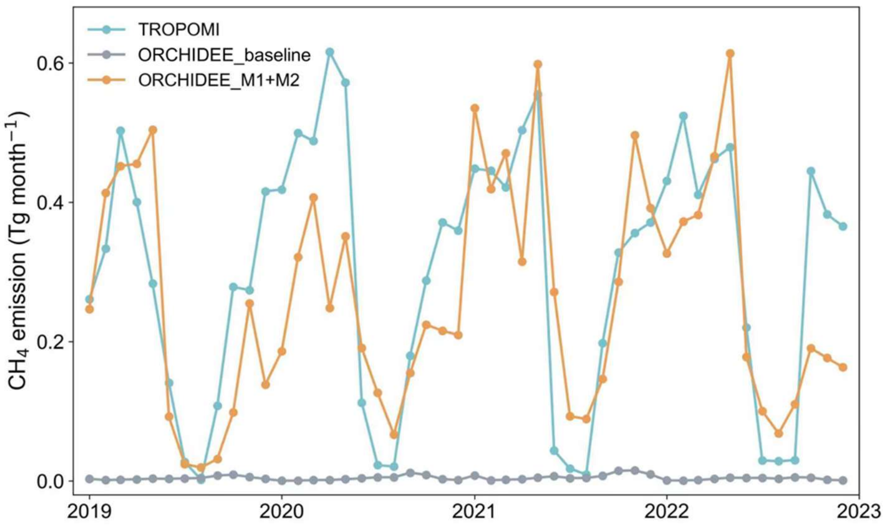

Fig. S7 Influence of AOD filtering on methane emissions.

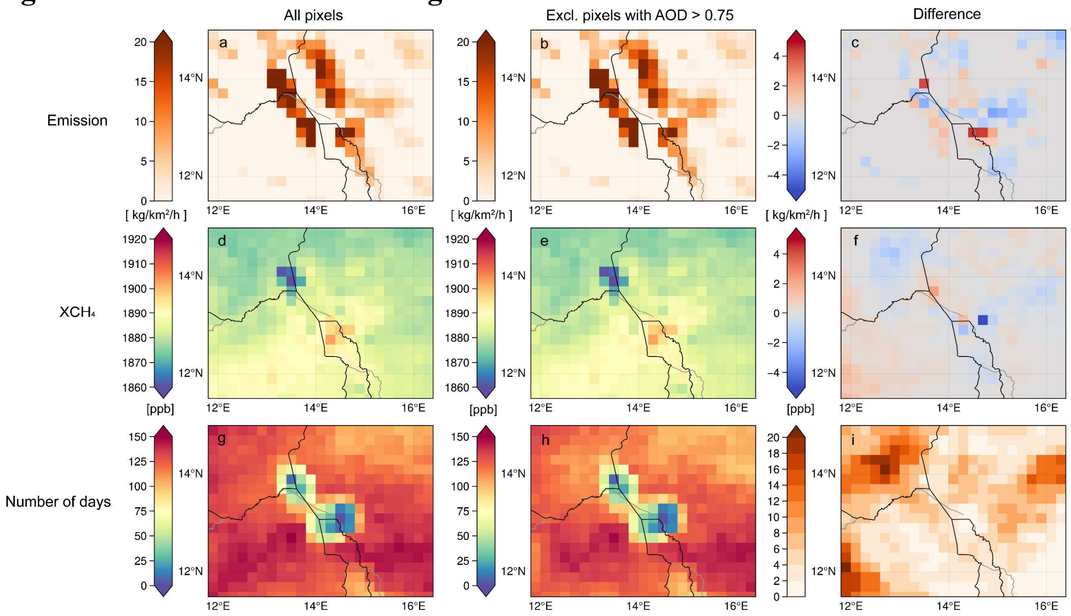

(a) Spatial distribution of the annual mean of methane emissions in 2020 based on all valid pixels. (b) Spatial distribution of the annual mean of methane emissions in 2020 after excluding pixels with AOD > 0.75. (c) Spatial distribution of the difference between a and b (a−b). (d)-(f) are similar to (a)-(c) but for results of XCH_4. (g)-(i) are similar to (a)-(c), but for the results of the number of valid days in 2020.

Fig. S8 Spatial distributions of the number of days with valid TROPOMI CH_4 observations for each month.

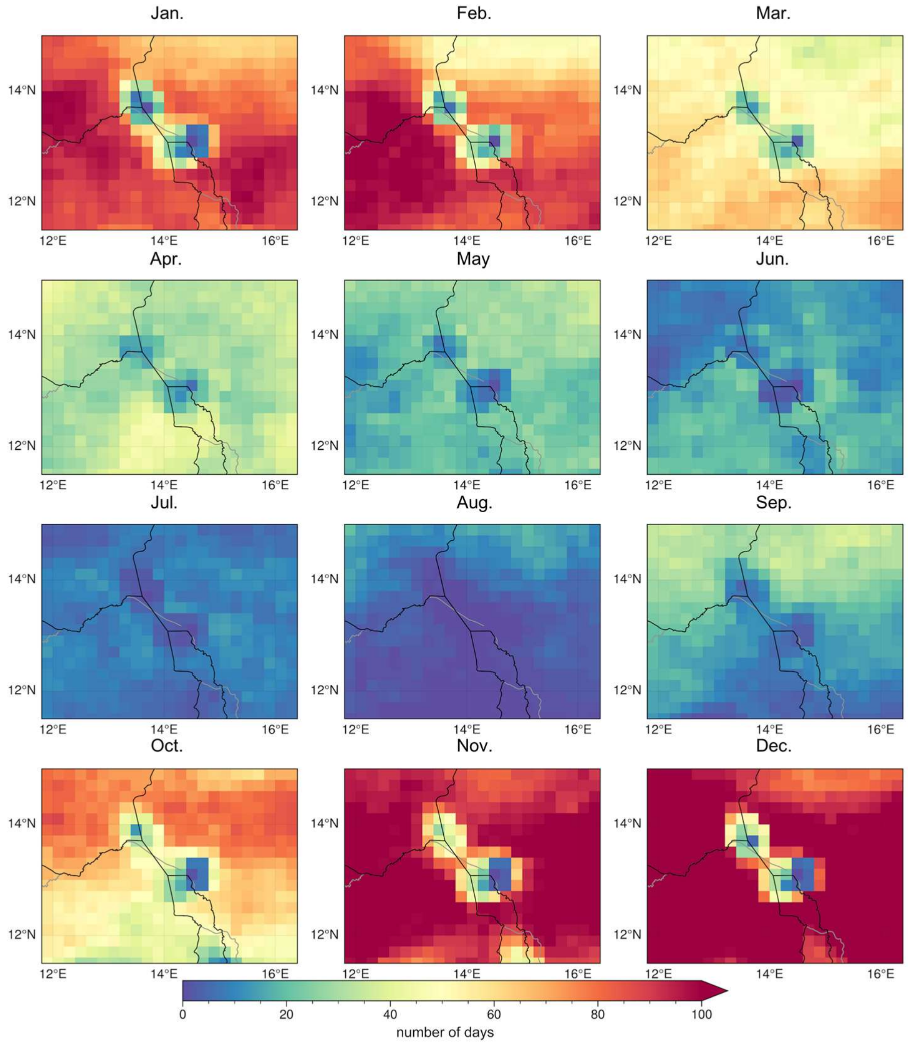

Total number of days per month summed over four years (2019–2022).

Fig. S9 The number of valid satellite observations over the Lake Chad region.

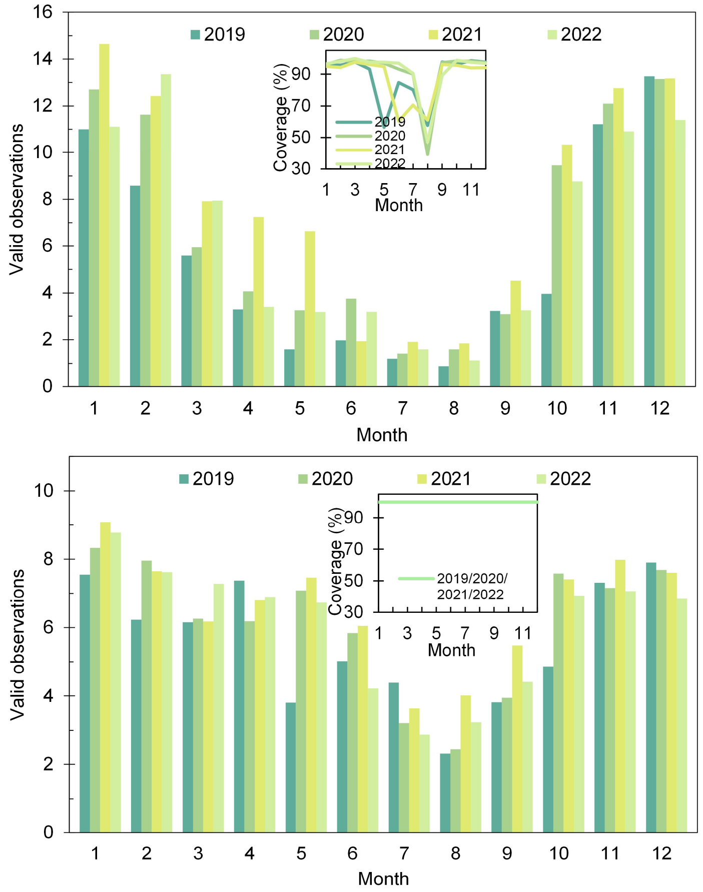

(a) for TROPOMI and (b) for Sentinel-2 in days per month. The insets show the monthly mean area coverage (%) for the whole region.

Fig. S10 Influence of regional background on methane emissions.

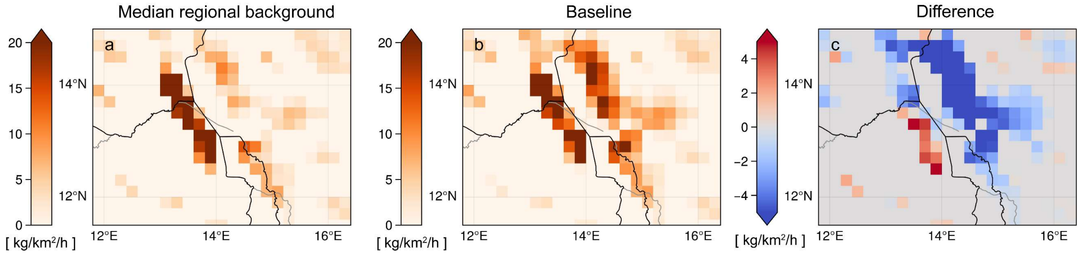

(a) Spatial distribution of the annual mean of methane emissions in 2020 based on setting a regional background for each grid cell as the median value of its surrounding 7×7 grid cells. (b) Spatial distribution of the annual mean of methane emissions in 2020 based on setting a regional background for each grid cell as the mean value of the lower 10 percent of its surrounding 7×7 grid cells. (c) Spatial distribution of the difference between a and b (a−b).

Fig. S11 CH_4 emission magnitude of the Lake Chad region.

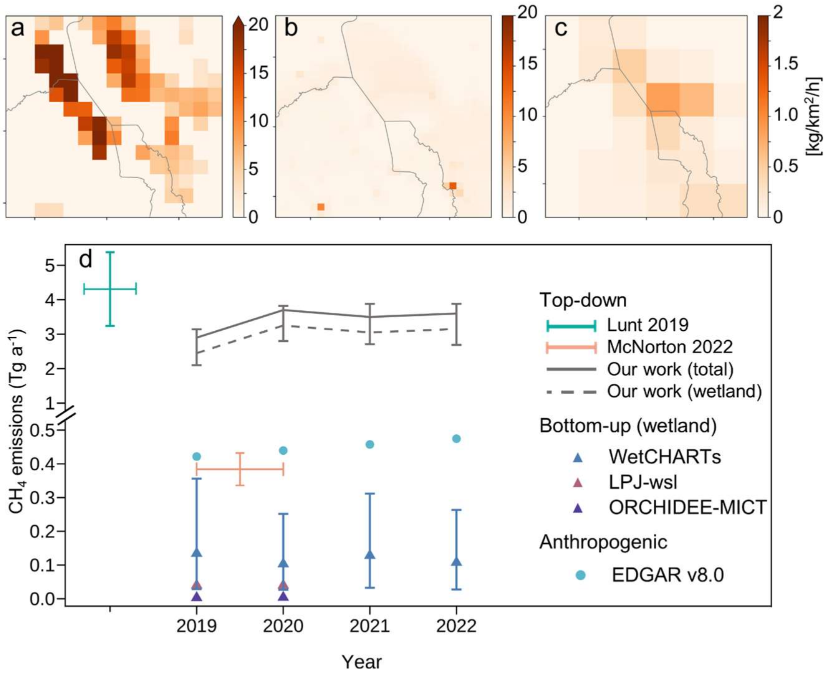

(a) Spatial distribution of TROPOMI-derived annual mean total methane emissions on a grid of 0.2° for 2019–2022. (b) Spatial distribution of EDGAR v8.0 emission estimates for anthropogenic sectors is shown for 2019–2022 (*75*). (c) Spatial distribution of WetCHARTs v1.3.3 bottom-up wetland emission estimates is shown for 2019–2022.08 (*29*). Note that the (a)-(c) have different scales. (d) Annual CH_4 emissions (Tg a^{-1}) for TROPOMI compared with literature estimates and bottom-up estimates. Retrieval uncertainties from TROPOMI incorporate sensitivities to missing data and local background calculation (see Supplementary Materials for details). WetCHARTs v1.3.3 includes uncertainty estimates derived from an 18-model ensemble.

Fig. S12 Flowchart showing how monthly water areas are derived from Sentinel-2 observations.

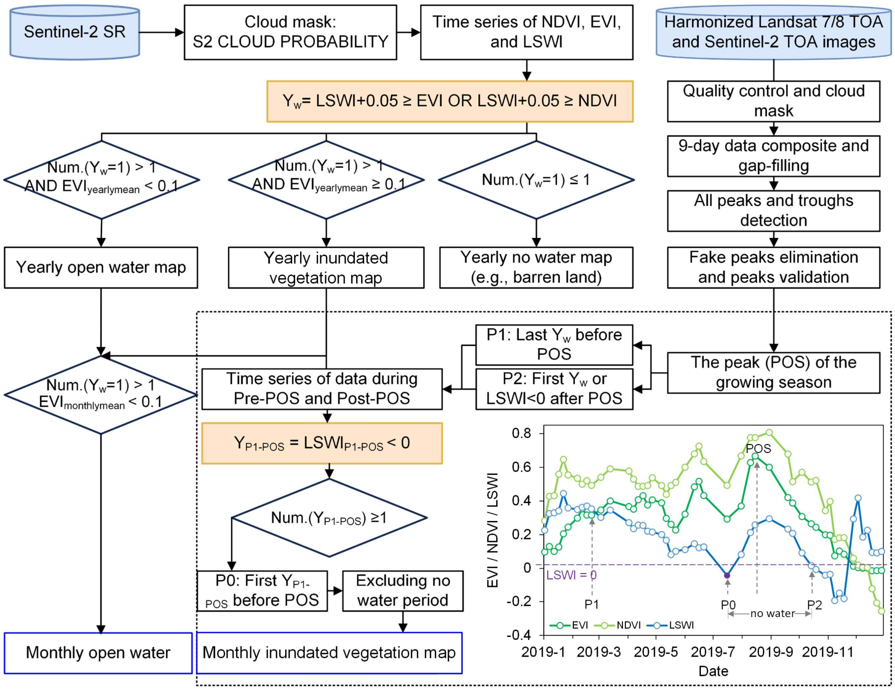

The lower-right figure shows the time series of EVI, NDVI, and LSWI for a single pixel (13°39'56"E, 13°46'46"N, 10×10m), demonstrating the classification of inundated vegetation during the closed canopy phase.

Fig. S13 CH_4 emissions–wetland inundation area dependencies (lines) derived from four-year monthly estimates (dots) without excluding data affected by poor-quality satellite observations.

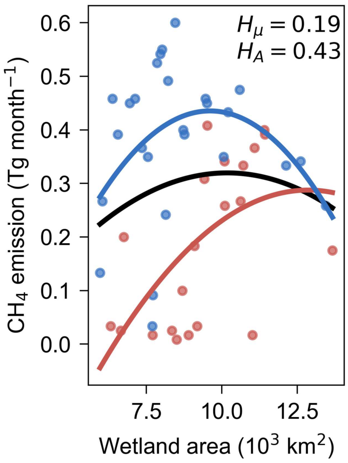

Fig. S14 Land cover map of the Lake Chad region (2019–2022) from GWL_FC30 (*95*).

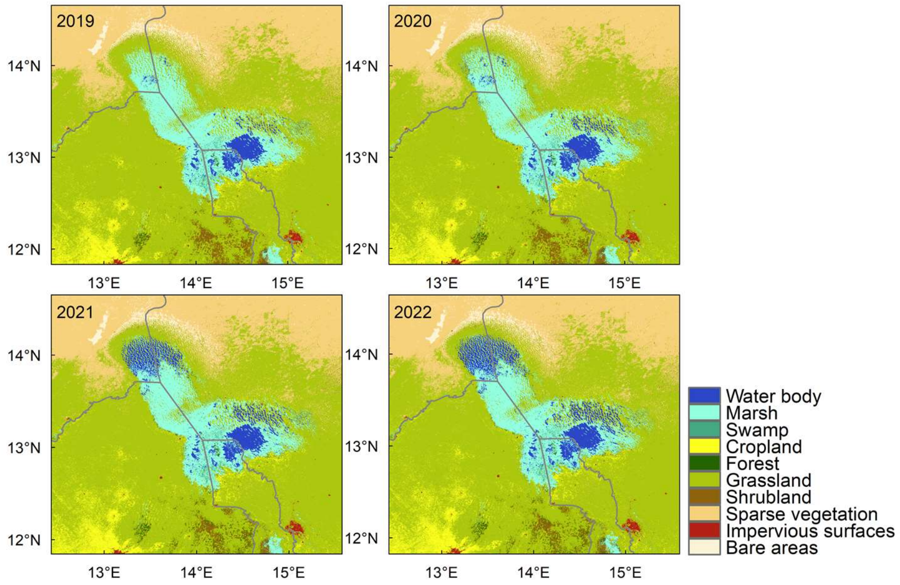

Fig. S15 Spatial distributions and frequency of occurrence of water over the Lake Chad region.

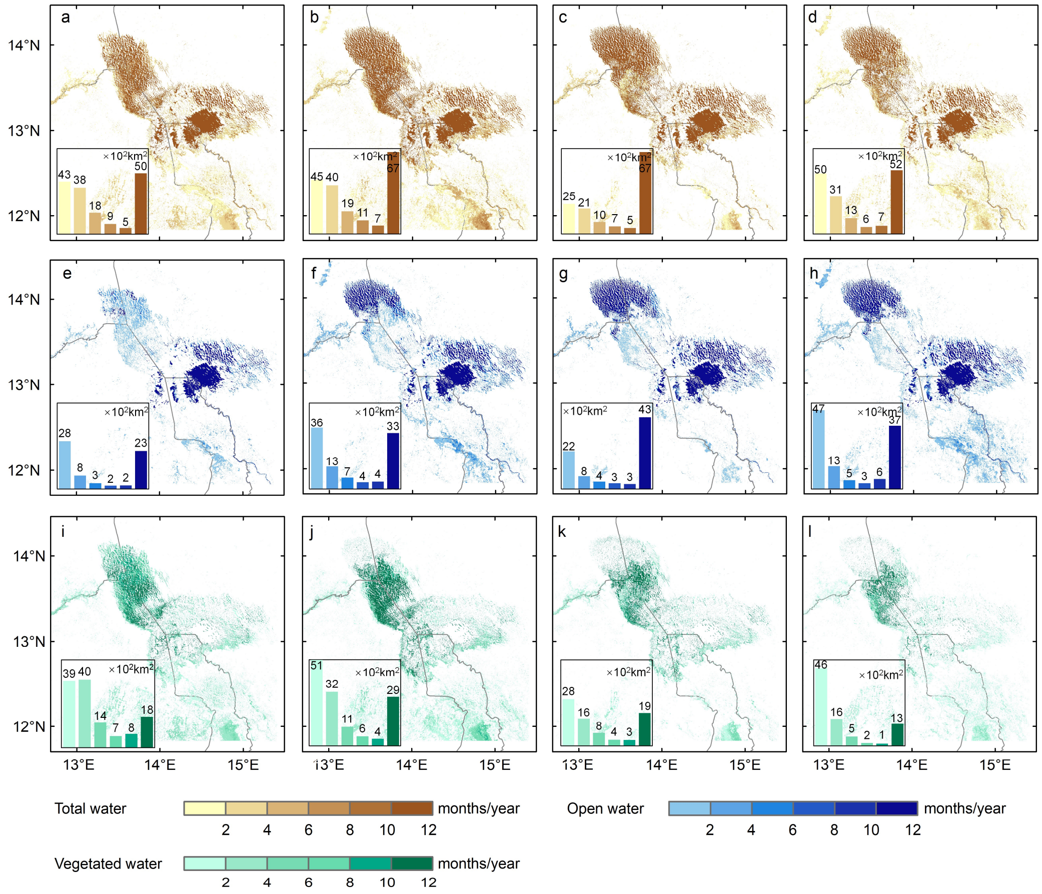

(a)-(d) Spatial distributions of total water (months/year) derived from Sentinel-2 observations for the years 2019, 2020, 2021, and 2022, respectively; (e)-(h) the same as (a)-(d) but for open water; (i)-(l) the same as (a)-(d) but for inundated vegetation. The insets show the frequency of occurrence of inundation area represented by total inundated areas (10^2 km^2) during 2, 4, 6, 8, 10, and 12 months per year, respectively.

Table S1. Cross-coefficient between monthly precipitation and monthly CH_4 emissions with different time lags (months) in the study area from 2019 to 2022.

	0	1	2	3	4	5	6	7	8	9	10	11	12
GPM	-0.79	-0.58	-0.17	0.07	0.17	0.26	0.36	**0.45**	0.43	0.30	-0.12	-0.50	-0.57
ERA5	-0.71	-0.44	-0.07	0.08	0.15	0.26	0.38	0.39	**0.43**	0.30	-0.27	-0.51	-0.55
MERRA2	-0.79	-0.57	-0.17	0.07	0.18	0.27	0.39	**0.48**	0.41	0.31	-0.14	-0.51	-0.59
TAMSAT	-0.81	-0.62	-0.22	0.04	0.17	0.29	0.42	**0.47**	0.47	0.34	-0.14	-0.50	-0.61

Note: The numbers 0-12 mean time-lag (months) of CH_4 emissions. Four monthly precipitation products derived from GPM, ERA5_LAND, MERRA-2, and TAMSAT. The most dominant cross correlations are bold.

Table S2. Cross-coefficient between monthly precipitation and monthly total water area with different time lags (months) in the study area from 2019 to 2022.

	0	1	2	3	4	5	6	7	8	9	10	11	12
GPM	-0.09	0.26	0.50	**0.60**	0.54	0.34	0.07	-0.13	-0.29	-0.40	-0.42	-0.31	-0.10
ERA5	-0.07	0.29	0.51	**0.58**	0.54	0.33	0.06	-0.15	-0.32	-0.42	-0.45	-0.33	-0.12
MERRA2	-0.02	0.27	0.44	**0.52**	0.47	0.23	0.01	-0.17	-0.31	-0.40	-0.40	-0.28	-0.03
TAMSAT	-0.13	0.22	0.47	**0.61**	0.59	0.37	0.10	-0.13	-0.31	-0.43	-0.45	-0.33	-0.07

Note: The numbers 0-12 mean time-lag (months) of total water area. Four monthly precipitation products derived from GPM, ERA5_LAND, MERRA-2, and TAMSAT. The most dominant cross correlations are bold.

Table S3. Uncertainty of methane monthly emission estimates from 2019 to 2022 based on sensitivity tests.

	AOD at 550 nm [a]	Regional background [b]	Uncertainty (Tg $month^{-1}$) [c]
Baseline	≤0.75	Mean of Lower 10 percentile	/
Test 1	All values	Mean of Lower 10 percentile	[-0.04, 0.13]
Test 2	≤0.75	Median value	[-0.2, 0.03]
Test 3 (missing values)	≤0.75	Mean of Lower 10 percentile	[0, 0.03]

[a] AOD value for each pixel is obtained based on the MODIS Level-2 10km aerosol product, MYD04_L2.006 (https://modaps.modaps.eosdis.nasa.gov/services/about/products/c6/MYD04_L2.html), and the CMAS EAC4 global hourly reanalysis dataset (https://ads.atmosphere.copernicus.eu/datasets/cams-global-reanalysis-eac4?tab=download). The methodology is followed by Liu *et al* (*66*).

[b] The domain of daily regional background for each grid cell is defined as its surrounding 3 by 3 grid cells (7×7=49 grid cells in total).

[c] The range represents the smallest to largest monthly sensitivity values occurring across the study period.

Table S4. Validation results of monthly open water and inundated vegetation maps by year.

Year	Ow OE	Ow CE	Iv OE	Iv CE	Ow classified as Iv	Iv classified as Ow	OA (water and non-water)	OA (Ow, Iv, non-water)
2019	0.11	0.09	0.26	0.16	0.09	0.09	0.95	0.92
2020	0.06	0.06	0.32	0.14	0.03	0.09	0.92	0.90
2021	0.04	0.10	0.29	0.07	0.02	0.12	0.94	0.93
2022	0.03	0.08	0.30	0.10	0.04	0.04	0.89	0.92

Ow: Open water, Iv: Inundated vegetation, OE: Omission Error, CE: Commission Error, OA: Overall Accuracy

REFERENCES

1. Intergovernmental Panel on Climate Change (IPCC), *Climate Change 2021 – The Physical Science Basis: Working Group I Contribution to the Sixth Assessment Report of the Intergovernmental Panel on Climate Change* (Cambridge Univ. Press, 2023); www.cambridge.org/core/product/415F29233B8BD19FB55F65E3DC67272B.

2. X. Lan, K. W. Thoning, E. J. Dlugokencky, Trends in globally-averaged CH4, N2O, and SF6 determined from NOAA Global Monitoring Laboratory measurements, version 2024–06, NOAA Global Monitoring Laboratory (2024); https://doi.org/10.15138/P8XG-AA10.

3. Z. Qu, D. J. Jacob, A. A. Bloom, J. R. Worden, R. J. Parker, H. Boesch, Inverse modeling of 2010–2022 satellite observations shows that inundation of the wet tropics drove the 2020–2022 methane surge. *Proc. Natl. Acad. Sci. U.S.A.* **121**, e2402730121 (2024).

4. Z. Qu, D. J. Jacob, Y. Zhang, L. Shen, D. J. Varon, X. Lu, T. Scarpelli, A. Bloom, J. Worden, R. J. Parker, Attribution of the 2020 surge in atmospheric methane by inverse analysis of GOSAT observations. *Environ. Res. Lett.* **17**, 094003 (2022).

5. H. Schaefer, S. E. M. Fletcher, C. Veidt, K. R. Lassey, G. W. Brailsford, T. M. Bromley, E. J. Dlugokencky, S. E. Michel, J. B. Miller, I. Levin, D. C. Lowe, R. J. Martin, B. H. Vaughn, J. W. C. White, A 21st-century shift from fossil-fuel to biogenic methane emissions indicated by $^{13}CH_4$. *Science* **352**, 80–84 (2016).

6. S. Peng, X. Lin, R. L. Thompson, Y. Xi, G. Liu, D. Hauglustaine, X. Lan, B. Poulter, M. Ramonet, M. Saunois, Y. Yin, Z. Zhang, B. Zheng, P. Ciais, Wetland emission and atmospheric sink changes explain methane growth in 2020. *Nature* **612**, 477–482 (2022).

7. E. Dowd, C. Wilson, M. P. Chipperfield, E. Gloor, A. Manning, R. Doherty, Decreasing seasonal cycle amplitude of methane in the northern high latitudes being driven by lower-latitude changes in emissions and transport. *Atmos. Chem. Phys.* **23**, 7363–7382 (2023).

8. G. Liu, L. Shen, P. Ciais, X. Lin, D. Hauglustaine, X. Lan, A. J. Turner, Y. Xi, Y. Zhu, S. Peng, Trends in the seasonal amplitude of atmospheric methane. *Nature* **641**, 660–665 (2025).

9. E. N. Koffi, P. Bergamaschi, R. Alkama, A. Cescatti, An observation-constrained assessment of the climate sensitivity and future trajectories of wetland methane emissions. *Sci. Adv.* **6**, eaay4444 (2020).

10. S. Kirschke, P. Bousquet, P. Ciais, M. Saunois, J. G. Canadell, E. J. Dlugokencky, P. Bergamaschi, D. Bergmann, D. R. Blake, L. Bruhwiler, P. Cameron-Smith, S. Castaldi, F. Chevallier, L. Feng, A. Fraser, M. Heimann, E. L. Hodson, S. Houweling, B. Josse, P. J. Fraser, P. B. Krummel, J.-F. Lamarque, R. L. Langenfelds, C. Le Quéré, V. Naik, S. O'Doherty, P. I. Palmer, I. Pison, D. Plummer, B. Poulter, R. G. Prinn, M. Rigby, B. Ringeval, M. Santini, M. Schmidt, D. T. Shindell, I. J. Simpson, R. Spahni, L. P. Steele, S. A. Strode, K. Sudo, S. Szopa, G. R. van der Werf, A. Voulgarakis, M. van Weele, R. F. Weiss, J. E. Williams, G. Zeng, Three decades of global methane sources and sinks. *Nat. Geosci.* **6**, 813–823 (2013).

11. A. J. Turner, C. Frankenberg, E. A. Kort, Interpreting contemporary trends in atmospheric methane. *Proc. Natl. Acad. Sci. U.S.A.* **116**, 2805–2813 (2019).

12. Z. Zhang, B. Poulter, A. F. Feldman, Q. Ying, P. Ciais, S. Peng, X. Li, Recent intensification of wetland methane feedback. *Nat. Clim. Change* **13**, 430–433 (2023).

13. H. Wang, J. Liu, M. Klaar, A. Chen, L. Gudmundsson, J. Holden, Anthropogenic climate change has influenced global river flow seasonality. *Science* **383**, 1009–1014 (2024).

14. L. Li, D. Long, Y. Wang, R. I. Woolway, Global dominance of seasonality in shaping lake-surface-extent dynamics. *Nature* **642**, 361–368 (2025).

15. R. J. Parker, H. Boesch, J. McNorton, E. Comyn-Platt, M. Gloor, C. Wilson, M. P. Chipperfield, G. D. Hayman, A. A. Bloom, Evaluating year-to-year anomalies in tropical wetland methane emissions using satellite CH4 observations. *Remote Sens. Environ.* **211**, 261–275 (2018).

16. Y. Yin, F. Chevallier, P. Ciais, P. Bousquet, M. Saunois, B. Zheng, J. Worden, A. A. Bloom, R. J. Parker, D. J. Jacob, E. J. Dlugokencky, C. Frankenberg, Accelerating methane growth

rate from 2010 to 2017: Leading contributions from the tropics and East Asia. *Atmos. Chem. Phys.* **21**, 12631–12647 (2021).

17. Z. Zhang, N. E. Zimmermann, A. Stenke, X. Li, E. L. Hodson, G. Zhu, C. Huang, B. Poulter, Emerging role of wetland methane emissions in driving 21st century climate change. *Proc. Natl. Acad. Sci. U.S.A.* **114**, 9647–9652 (2017).

18. Z. Zhang, N. E. Zimmermann, L. Calle, G. Hurtt, A. Chatterjee, B. Poulter, Enhanced response of global wetland methane emissions to the 2015–2016 El Niño-Southern Oscillation event. *Environ. Res. Lett.* **13**, 074009 (2018).

19. A. A. Bloom, K. W. Bowman, M. Lee, A. J. Turner, R. Schroeder, J. R. Worden, R. Weidner, K. C. McDonald, D. J. Jacob, A global wetland methane emissions and uncertainty dataset for atmospheric chemical transport models (WetCHARTs version 1.0). *Geosci. Model Dev.* **10**, 2141–2156 (2017).

20. Y. Xi, S. Peng, A. Ducharne, P. Ciais, T. Gumbricht, C. Jimenez, B. Poulter, C. Prigent, C. Qiu, M. Saunois, Z. Zhang, Gridded maps of wetlands dynamics over mid-low latitudes for 1980–2020 based on TOPMODEL. *Sci. Data* **9**, 347 (2022).

21. M. Li, E. A. Kort, A. A. Bloom, D. Wu, G. Plant, C. Gerlein-Safdi, T. Pu, Underestimated dry season methane emissions from wetlands in the Pantanal. *Environ. Sci. Technol.* **58**, 3278–3287 (2024).

22. C. Helfter, M. Gondwe, M. Murray-Hudson, A. Makati, M. F. Lunt, P. I. Palmer, U. Skiba, Phenology is the dominant control of methane emissions in a tropical non-forested wetland. *Nat. Commun.* **13**, 133 (2022).

23. S. H. Knox, S. Bansal, G. McNicol, K. Schafer, C. Sturtevant, M. Ueyama, A. C. Valach, D. Baldocchi, K. Delwiche, A. R. Desai, E. Euskirchen, J. Liu, A. Lohila, A. Malhotra, L. Melling, W. Riley, B. R. K. Runkle, J. Turner, R. Vargas, Q. Zhu, T. Alto, E. Fluet-Chouinard, M. Goeckede, J. R. Melton, O. Sonnentag, T. Vesala, E. Ward, Z. Zhang, S. Feron, Z. Ouyang, P. Alekseychik, M. Aurela, G. Bohrer, D. I. Campbell, J. Chen, H. Chu, H. J. Dalmagro, J. P. Goodrich, P. Gottschalk, T. Hirano, H. Iwata, G. Jurasinski, M. Kang, F.

Koebsch, I. Mammarella, M. B. Nilsson, K. Ono, M. Peichl, O. Peltola, Y. Ryu, T. Sachs, A. Sakabe, J. P. Sparks, E. S. Tuittila, G. L. Vourlitis, G. X. Wong, L. Windham-Myers, B. Poulter, R. B. Jackson, Identifying dominant environmental predictors of freshwater wetland methane fluxes across diurnal to seasonal time scales. *Glob. Change Biol.* **27**, 3582–3604 (2021).

24. S. Feron, A. Malhotra, S. Bansal, E. Fluet-Chouinard, G. McNicol, S. H. Knox, K. B. Delwiche, R. R. Cordero, Z. Ouyang, Z. Zhang, B. Poulter, R. B. Jackson, Recent increases in annual, seasonal, and extreme methane fluxes driven by changes in climate and vegetation in boreal and temperate wetland ecosystems. *Glob. Change Biol.* **30**, e17131 (2024).

25. K.-Y. Chang, W. J. Riley, S. H. Knox, R. B. Jackson, G. McNicol, B. Poulter, M. Aurela, D. Baldocchi, S. Bansal, G. Bohrer, D. I. Campbell, A. Cescatti, H. Chu, K. B. Delwiche, A. R. Desai, E. Euskirchen, T. Friborg, M. Goeckede, M. Helbig, K. S. Hemes, T. Hirano, H. Iwata, M. Kang, T. Keenan, K. W. Krauss, A. Lohila, I. Mammarella, B. Mitra, A. Miyata, M. B. Nilsson, A. Noormets, W. C. Oechel, D. Papale, M. Peichl, M. L. Reba, J. Rinne, B. R. K. Runkle, Y. Ryu, T. Sachs, K. V. R. Schäfer, H. P. Schmid, N. Shurpali, O. Sonnentag, A. C. I. Tang, M. S. Torn, C. Trotta, E.-S. Tuittila, M. Ueyama, R. Vargas, T. Vesala, L. Windham-Myers, Z. Zhang, D. Zona, Substantial hysteresis in emergent temperature sensitivity of global wetland CH4 emissions. *Nat. Commun.* **12**, 2266 (2021).

26. S. Pandey, S. Houweling, A. Lorente, T. Borsdorff, M. Tsivlidou, A. A. Bloom, B. Poulter, Z. Zhang, I. Aben, Using satellite data to identify the methane emission controls of South Sudan's wetlands. *Biogeosciences* **18**, 557–572 (2021).

27. M. F. Lunt, P. I. Palmer, A. Lorente, T. Borsdorff, J. Landgraf, R. J. Parker, H. Boesch, Rain-fed pulses of methane from East Africa during 2018–2019 contributed to atmospheric growth rate. *Environ. Res. Lett.* **16**, 024021 (2021).

28. M. K. Vanderhoof, L. Alexander, J. Christensen, K. Solvik, P. Nieuwlandt, M. Sagehorn, High-frequency time series comparison of Sentinel-1 and Sentinel-2 satellites for mapping open and vegetated water across the United States (2017–2021). *Remote Sens. Environ.* **288**, 113498 (2023).

29. A. A. Bloom, K. W. Bowman, M. Lee, A. J. Turner, R. Schroeder, J. R. Worden, R. J. Weidner, K. C. McDonald, D. J. Jacob, CMS: Global 0.5-deg Wetland Methane Emissions and Uncertainty (WetCHARTs v1.3.3), version 1.3.3, ORNL Distributed Active Archive Center (2024); https://doi.org/10.3334/ORNLDAAC/2346.

30. A. V. Borges, L. Deirmendjian, S. Bouillon, W. Okello, T. Lambert, F. A. E. Roland, V. F. Razanamahandry, N. R. G. Voarintsoa, F. Darchambeau, I. A. Kimirei, J.-P. Descy, G. H. Allen, C. Morana, Greenhouse gas emissions from African lakes are no longer a blind spot. *Sci. Adv.* **8**, eabi8716 (2022).

31. S. R. Pangala, A. Enrich-Prast, L. S. Basso, R. B. Peixoto, D. Bastviken, E. R. C. Hornibrook, L. V. Gatti, H. Marotta, L. S. B. Calazans, C. M. Sakuragui, W. R. Bastos, O. Malm, E. Gloor, J. B. Miller, V. Gauci, Large emissions from floodplain trees close the Amazon methane budget. *Nature* **552**, 230–234 (2017).

32. S. Bansal, M. Post van der Burg, R. R. Fern, J. W. Jones, R. Lo, O. P. McKenna, B. A. Tangen, Z. Zhang, R. A. Gleason, Large increases in methane emissions expected from North America's largest wetland complex. *Sci. Adv.* **9**, eade1112 (2023).

33. I. Forbrich, T. Yazbeck, B. Sulman, T. H. Morin, A. C. I. Tang, G. Bohrer, Three decades of wetland methane surface flux modeling by Earth system models-advances, applications, and challenges. *J. Geophys. Res. Biogeosci.* **129**, e2023JG007915 (2024).

34. H. Qian, X. Zhu, S. Huang, B. Linquist, Y. Kuzyakov, R. Wassmann, K. Minamikawa, M. Martinez-Eixarch, X. Yan, F. Zhou, B. O. Sander, W. Zhang, Z. Shang, J. Zou, X. Zheng, G. Li, Z. Liu, S. Wang, Y. Ding, K. J. van Groenigen, Y. Jiang, Greenhouse gas emissions and mitigation in rice agriculture. *Nat. Rev. Earth Environ.* **4**, 716–732 (2023).

35. R. Dommain, J. Couwenberg, P. H. Glaser, H. Joosten, I. N. N. Suryadiputra, Carbon storage and release in Indonesian peatlands since the last deglaciation. *Quat. Sci. Rev.* **97**, 1–32 (2014).

36. S. Cui, P. Liu, H. Guo, C. K. Nielsen, J. W. M. Pullens, Q. Chen, L. Pugliese, S. Wu, Wetland hydrological dynamics and methane emissions. *Commun. Earth Environ.* **5**, 470 (2024).

37. S. D. Bridgham, H. Cadillo-Quiroz, J. K. Keller, Q. Zhuang, Methane emissions from wetlands: Biogeochemical, microbial, and modeling perspectives from local to global scales. *Glob. Change Biol.* **19**, 1325–1346 (2013).

38. H. Chen, X. Xu, C. Fang, B. Li, M. Nie, Differences in the temperature dependence of wetland CO_2 and CH_4 emissions vary with water table depth. *Nat. Clim. Change* **11**, 766–771 (2021).

39. C. Gerlein-Safdi, A. A. Bloom, G. Plant, E. A. Kort, C. S. Ruf, Improving representation of tropical wetland methane emissions with CYGNSS inundation maps. *Global Biogeochem. Cycles* **35**, e2020GB006890 (2021).

40. Y. Xiong, E. A. Kort, A. A. Bloom, C. Gerlein-Safdi, T. Pu, E. Bilir, Limited evidence that tropical inundation and precipitation powered the 2020–2022 methane surge. *Commun. Earth Environ.* **6**, 450 (2025).

41. M. Gloor, L. V. Gatti, C. Wilson, R. J. Parker, H. Boesch, E. Popa, M. P. Chipperfield, B. Poulter, Z. Zhang, L. Basso, J. Miller, J. McNorton, C. Jimenez, C. Prigent, Large methane emissions from the Pantanal during rising water-levels revealed by regularly measured lower troposphere CH_4 profiles. *Global Biogeochem. Cycles* **35**, e2021GB006964 (2021).

42. A. C. I. Tang, G. Bohrer, A. Malhotra, J. Missik, F. Machado-Silva, I. Forbrich, Rising water levels and vegetation shifts drive substantial reductions in methane emissions and carbon dioxide uptake in a great lakes coastal freshwater wetland. *Glob. Change Biol.* **31**, e70053 (2025).

43. Z. Zhang, B. Poulter, J. R. Melton, W. J. Riley, G. H. Allen, D. J. Beerling, P. Bousquet, J. G. Canadell, E. Fluet-Chouinard, P. Ciais, N. Gedney, P. O. Hopcroft, A. Ito, R. B. Jackson, A. K. Jain, K. Jensen, F. Joos, T. Kleinen, S. H. Knox, T. Li, X. Li, X. Liu, K. McDonald, G. McNicol, P. A. Miller, J. Müller, P. K. Patra, C. Peng, S. Peng, Z. Qin, R. M. Riggs, M. Saunois, Q. Sun, H. Tian, X. Xu, Y. Yao, Y. Xi, W. Zhang, Q. Zhu, Q. Zhu, Q. Zhuang, Ensemble estimates of global wetland methane emissions over 2000–2020. *Biogeosciences* **22**, 305–321 (2025).

44. Q. Zhu, C. Peng, P. Ciais, H. Jiang, J. Liu, P. Bousquet, S. Li, J. Chang, X. Fang, X. Zhou, H. Chen, S. Liu, G. Lin, P. Gong, M. Wang, H. Wang, W. Xiang, J. Chen, Interannual variation in methane emissions from tropical wetlands triggered by repeated El Niño Southern Oscillation. *Glob. Change Biol.* **23**, 4706–4716 (2017).

45. X. Xu, F. Yuan, P. J. Hanson, S. D. Wullschleger, P. E. Thornton, W. J. Riley, X. Song, D. E. Graham, C. Song, H. Tian, Reviews and syntheses: Four decades of modeling methane cycling in terrestrial ecosystems. *Biogeosciences* **13**, 3735–3755 (2016).

46. P. Zeiger, F. Frappart, J. Darrozes, C. Prigent, C. Jiménez, L. Bourrel, Weekly mapping of surface water extent in the intertropical wetlands using spaceborne GNSS reflectometry. *J. Hydrol.* **626**, 130305 (2023).

47. D. L. Engle, J. M. Melack, R. D. Doyle, T. R. Fisher, High rates of net primary production and turnover of floating grasses on the Amazon floodplain: Implications for aquatic respiration and regional CO_2 flux. *Glob. Change Biol.* **14**, 369–381 (2008).

48. T. S. F. Silva, J. M. Melack, E. M. L. M. Novo, Responses of aquatic macrophyte cover and productivity to flooding variability on the Amazon floodplain. *Glob. Change Biol.* **19**, 3379–3389 (2013).

49. G. Oakes, A. Hardy, P. Bunting, RadWet: An improved and transferable mapping of open water and inundated vegetation using Sentinel-1. *Remote Sens.* **15**, 1705 (2023).

50. C. Hübinger, E. Fluet-Chouinard, D. Escobar, F. Jaramillo, Exploring the potential of using L-band InSAR for mapping flooded vegetation in tropical wetlands. *Remote Sens. Environ.* **332**, 115086 (2026).

51. "Sentinel-5 data products" (European Space Agency, 2025); https://sentinels.copernicus.eu/missions/sentinel-5/data-products.

52. Y. Zhang, R. Gautam, S. Pandey, M. Omara, J. D. Maasakkers, P. Sadavarte, D. Lyon, H. Nesser, M. P. Sulprizio, D. J. Varon, R. Zhang, S. Houweling, D. Zavala-Araiza, R. A. Alvarez, A. Lorente, S. P. Hamburg, I. Aben, D. J. Jacob, Quantifying methane emissions

from the largest oil-producing basin in the United States from space. *Sci. Adv.* **6**, eaaz5120 (2020).

53. M. Liu, R. van der A, M. van Weele, H. Eskes, X. Lu, P. Veefkind, J. de Laat, H. Kong, J. Wang, J. Sun, J. Ding, Y. Zhao, H. Weng, A new divergence method to quantify methane emissions using observations of Sentinel-5P TROPOMI. *Geophys. Res. Lett.* **48**, e2021GL094151 (2021).

54. C. E. Ndehedehe, V. G. Ferreira, A. O. Onojeghuo, N. O. Agutu, E. Emengini, A. Getirana, Influence of global climate on freshwater changes in Africa's largest endorheic basin using multi-scaled indicators. *Sci. Total Environ.* **737**, 139643 (2020).

55. C. M. Taylor, C. Prigent, S. J. Dadson, Mesoscale rainfall patterns observed around wetlands in sub-Saharan Africa. *Q. J. Roy. Meteorol. Soc.* **144**, 2118–2132 (2018).

56. M. Leblanc, J. Lemoalle, J. C. Bader, S. Tweed, L. Mofor, Thermal remote sensing of water under flooded vegetation: New observations of inundation patterns for the 'Small' Lake Chad. *J. Hydrol.* **404**, 87–98 (2011).

57. "Inter-Tropical Convergence Zone" (National Oceanic and Atmospheric Administration (NOAA), 2023); www.noaa.gov/jetstream/tropical/convergence-zone.

58. B. Pham-Duc, F. Sylvestre, F. Papa, F. Frappart, C. Bouchez, J.-F. Crétaux, The Lake Chad hydrology under current climate change. *Sci. Rep.* **10**, 5498 (2020).

59. "LAKE CHAD BASIN: Impact of extreme weather and climate events on livelihoods and food security" (Assessment Capacities Project, 2022); www.acaps.org/fileadmin/Data_Product/Main_media/20220816_acaps_thematic_report_global_analysis_team_lake_chad_basin_0.pdf.

60. M. F. Lunt, P. I. Palmer, L. Feng, C. M. Taylor, H. Boesch, R. J. Parker, An increase in methane emissions from tropical Africa between 2010 and 2016 inferred from satellite data. *Atmos. Chem. Phys.* **19**, 14721–14740 (2019).

61. J. McNorton, N. Bousserez, A. Agusti-Panareda, G. Balsamo, L. Cantarello, R. Engelen, V. Huijnen, A. Inness, Z. Kipling, M. Parrington, R. Ribas, Quantification of methane emissions from hotspots and during COVID-19 using a global atmospheric inversion. *Atmos. Chem. Phys.* **22**, 5961–5981 (2022).

62. J. P. Veefkind, I. Aben, K. McMullan, H. Förster, J. de Vries, G. Otter, J. Claas, H. J. Eskes, J. F. de Haan, Q. Kleipool, M. van Weele, O. Hasekamp, R. Hoogeveen, J. Landgraf, R. Snel, P. Tol, P. Ingmann, R. Voors, B. Kruizinga, R. Vink, H. Visser, P. F. Levelt, TROPOMI on the ESA Sentinel-5 Precursor: A GMES mission for global observations of the atmospheric composition for climate, air quality and ozone layer applications. *Remote Sens. Environ.* **120**, 70–83 (2012).

63. A. Lorente, T. Borsdorff, A. Butz, O. Hasekamp, J. aan de Brugh, A. Schneider, L. Wu, F. Hase, R. Kivi, D. Wunch, D. F. Pollard, K. Shiomi, N. M. Deutscher, V. A. Velazco, C. M. Roehl, P. O. Wennberg, T. Warneke, J. Landgraf, Methane retrieved from TROPOMI: Improvement of the data product and validation of the first 2 years of measurements. *Atmos. Meas. Tech.* **14**, 665–684 (2021).

64. O. Schneising, M. Buchwitz, J. Hachmeister, S. Vanselow, M. Reuter, M. Buschmann, H. Bovensmann, J. P. Burrows, Advances in retrieving XCH4and XCO from Sentinel-5 Precursor: Improvements in the scientific TROPOMI/WFMD algorithm. *Atmos. Meas. Tech.* **16**, 669–694 (2023).

65. O. Schneising, M. Buchwitz, M. Reuter, H. Bovensmann, J. P. Burrows, T. Borsdorff, N. M. Deutscher, D. G. Feist, D. W. T. Griffith, F. Hase, C. Hermans, L. T. Iraci, R. Kivi, J. Landgraf, I. Morino, J. Notholt, C. Petri, D. F. Pollard, S. Roche, K. Shiomi, K. Strong, R. Sussmann, V. A. Velazco, T. Warneke, D. Wunch, A scientific algorithm to simultaneously retrieve carbon monoxide and methane from TROPOMI onboard Sentinel-5 Precursor. *Atmos. Meas. Tech.* **12**, 6771–6802 (2019).

66. M. Liu, R. van der A, M. van Weele, L. Bryan, H. Eskes, P. Veefkind, Y. Liu, X. Lin, J. de Laat, J. Ding, Current potential of CH_4 emission estimates using TROPOMI in the Middle East. *EGUsphere* **17**, 5261–5277 (2024).

67. Naval Research Laboratory, University of North Dakota, MODIS/Aqua Value-added Aerosol Optical Depth, version 6.1NRT, NASA LANCE MODIS at the MODAPS (2017); https://doi.org/10.5067/MODIS/MCDAODHD.NRT.061.

68. S. Beirle, C. Borger, S. Dörner, A. Li, Z. Hu, F. Liu, Y. Wang, T. Wagner, Pinpointing nitrogen oxide emissions from space. *Sci. Adv.* **5**, eaax9800 (2019).

69. Y. Chen, R. J. van der A, J. Ding, H. Eskes, J. E. Williams, N. Theys, A. Tsikerdekis, P. F. Levelt, SO_2 emissions derived from TROPOMI observations over India using a flux-divergence method with variable lifetimes. *Atmos. Chem. Phys.* **25**, 1851–1868 (2025).

70. J. P. Veefkind, R. Serrano-Calvo, J. de Gouw, B. Dix, O. Schneising, M. Buchwitz, J. Barré, R. J. van der A, M. Liu, P. F. Levelt, Widespread frequent methane emissions from the oil and gas industry in the Permian basin. *J. Geophys. Res. Atmos.* **128**, e2022JD037479 (2023).

71. A. Inness, M. Ades, A. Agustí-Panareda, J. Barré, A. Benedictow, A. M. Blechschmidt, J. J. Dominguez, R. Engelen, H. Eskes, J. Flemming, V. Huijnen, L. Jones, Z. Kipling, S. Massart, M. Parrington, V. H. Peuch, M. Razinger, S. Remy, M. Schulz, M. Suttie, The CAMS reanalysis of atmospheric composition. *Atmos. Chem. Phys.* **19**, 3515–3556 (2019).

72. X. Lan, J. W. Mund, A. M. Crotwell, K. W. Thoning, E. Moglia, M. Madronich, K. Baugh, G. Petron, M. J. Crotwell, D. Neff, S. Wolter, T. Mefford, S. DeVogel, Atmospheric Methane Dry Air Mole Fractions from the NOAA GML Carbon Cycle Cooperative Global Air Sampling Network, 1983–2023, version 2024-07-30, NOAA Global Monitoring Laboratory (2024); https://doi.org/10.15138/VNCZ-M766.

73. L. Bryan, "*The Flux Divergence Method Applied to Nitrogen Emissions in The Netherlands*", Master thesis, Delft University of Technology, Delft, the Netherlands (2022).

74. Z. Chen, D. J. Jacob, R. Gautam, M. Omara, R. N. Stavins, R. C. Stowe, H. Nesser, M. P. Sulprizio, A. Lorente, D. J. Varon, X. Lu, L. Shen, Z. Qu, D. C. Pendergrass, S. Hancock, Satellite quantification of methane emissions and oil–gas methane intensities from individual countries in the Middle East and North Africa: Implications for climate action. *Atmos. Chem. Phys.* **23**, 5945–5967 (2023).

75. M. Crippa, D. Guizzardi, E. Schaaf, F. Monforti-Ferrario, R. Quadrelli, A. Risquez Martin, S. Rossi, E. Vignati, M. Muntean, J. Brandao De Melo, D. Oom, F. Pagani, M. Banja, P. Taghavi-Moharamli, J. Köykkä, G. Grassi, A. Branco, J. San-Miguel, "GHG emissions of all world countries" (JRC134504, Publications Office of the European Union, 2023).

76. Z. Zhang, N. E. Zimmermann, J. O. Kaplan, B. Poulter, Modeling spatiotemporal dynamics of global wetlands: Comprehensive evaluation of a new sub-grid TOPMODEL parameterization and uncertainties. *Biogeosciences* **13**, 1387–1408 (2016).

77. X. Xiao, S. Boles, J. Liu, D. Zhuang, S. Frolking, C. Li, W. Salas, B. Moore, Mapping paddy rice agriculture in southern China using multi-temporal MODIS images. *Remote Sens. Environ.* **95**, 480–492 (2005).

78. X. Xiao, S. Boles, S. Frolking, C. Li, J. Y. Babu, W. Salas, B. Moore, Mapping paddy rice agriculture in South and Southeast Asia using multi-temporal MODIS images. *Remote Sens. Environ.* **100**, 95–113 (2006).

79. J. Yang, J. Dong, X. Xiao, J. Dai, C. Wu, J. Xia, G. Zhao, M. Zhao, Z. Li, Y. Zhang, Q. Ge, Divergent shifts in peak photosynthesis timing of temperate and alpine grasslands in China. *Remote Sens. Environ.* **233**, 111395 (2019).

80. M. Santa, "Planet imagery product specifications" (Planet Labs Inc., 2019); http://assets.planet.com/docs/combined-imagery-product-spec-april-2019.pdf.

81. C. Schwatke, D. Dettmering, W. Bosch, F. Seitz, DAHITI – An innovative approach for estimating water level time series over inland waters using multi-mission satellite altimetry. *Hydrol. Earth Syst. Sci.* **19**, 4345–4364 (2015).

82. D. Wiese, GRACE monthly global water mass grids NETCDF RELEASE 6.1, version 3.0, Physical Oceanography Distributed Active Archive Center (PO. DAAC) (2015); https://doi.org/10.5067/TEMSC-3MJ63.

83. Z. Zhang, E. Fluet-Chouinard, K. Jensen, K. McDonald, G. Hugelius, T. Gumbricht, M. Carroll, C. Prigent, A. Bartsch, B. Poulter, Development of the global dataset of Wetland

Area and Dynamics for Methane Modeling (WAD2M). *Earth Syst. Sci. Data* **13**, 2001–2023 (2021).

84. J.-F. Pekel, A. Cottam, N. Gorelick, A. S. Belward, High-resolution mapping of global surface water and its long-term changes. *Nature* **540**, 418–422 (2016).

85. T. Pu, C. Gerlein-Safdi, Y. Xiong, M. Li, E. A. Kort, A. A. Bloom, Berkeley-RWAWC: A new CYGNSS-based watermask unveils unique observations of seasonal dynamics in the tropics. *Water Resour. Res.* **60**, e2024WR037060 (2024).

86. Integration and Application Network, University of Maryland Center for Environmental Science. IAN Symbol and Image Libraries, https://ian.umces.edu/media-library/ [accessed 1 April 2025].

87. NASA Jet Propulsion Laboratory, VISIONS: The EMIT Open Data Portal, https://earth.jpl.nasa.gov/emit/data/data-portal/Greenhouse-Gases/ [accessed 1 March 2025].

88. Global Energy Monitor, Global Oil and Gas Plant Tracker, https://globalenergymonitor.org/projects/global-oil-gas-plant-tracker/tracker-map/ [accessed 1 March 2025].

89. T. Amole, A. Augustine, M. Balehegn, A. T. Adesogoan, Livestock feed resources in the West African Sahel. *Agron. J.* **114**, 26–45 (2022).

90. G. X. Gbenou, M. H. Assouma, D. Bastianelli, T. Kiendrebeogo, L. Bonnal, N. Zampaligre, B. Bois, S. Sanogo, O. Sib, C. Martin, L. H. Dossa, Enteric methane emissions from zebu cattle are influenced by seasonal variations in rangeland fodder quality and intake. *Animal* **18**, 101320 (2024).

91. M. P. Fortnam, J. A. Oguntola, "Global International Waters Assessment: Lake Chad Basin, GIWA Regional assessment 43" (1651–9401, University of Kalmar, Kalmar, Sweden, 2004); www.droughtmanagement.info/literature/UNEP_lake_chad_basin_2004.pdf.

92. S. Vanselow, O. Schneising, M. Buchwitz, M. Reuter, H. Bovensmann, H. Boesch, J. P. Burrows, Automated detection of regions with persistently enhanced methane concentrations using Sentinel-5 Precursor satellite data. *Atmos. Chem. Phys.* **24**, 10441–10473 (2024).

93. M. Saunois, A. Martinez, B. Poulter, Z. Zhang, P. Raymond, P. Regnier, J. G. Canadell, R. B. Jackson, P. K. Patra, P. Bousquet, P. Ciais, E. J. Dlugokencky, X. Lan, G. H. Allen, D. Bastviken, D. J. Beerling, D. A. Belikov, D. R. Blake, S. Castaldi, M. Crippa, B. R. Deemer, F. Dennison, G. Etiope, N. Gedney, L. Höglund-Isaksson, M. A. Holgerson, P. O. Hopcroft, G. Hugelius, A. Ito, A. K. Jain, R. Janardanan, M. S. Johnson, T. Kleinen, P. Krummel, R. Lauerwald, T. Li, X. Liu, K. C. McDonald, J. R. Melton, J. Mühle, J. Müller, F. Murguia-Flores, Y. Niwa, S. Noce, S. Pan, R. J. Parker, C. Peng, M. Ramonet, W. J. Riley, G. Rocher-Ros, J. A. Rosentreter, M. Sasakawa, A. Segers, S. J. Smith, E. H. Stanley, J. Thanwerdas, H. Tian, A. Tsuruta, F. N. Tubiello, T. S. Weber, G. van der Werf, D. E. Worthy, Y. Xi, Y. Yoshida, W. Zhang, B. Zheng, Q. Zhu, Q. Zhu, Q. Zhuang, Global Methane Budget 2000-2020. *Earth Syst. Sci. Data Discuss.* **2024**, 1–147 (2024).

94. Copernicus Sentinel-2 (processed by ESA), MSI Level-2A BOA Reflectance Product, Collection 1, European Space Agency (2021); https://doi.org/10.5270/S2_-znk9xsj.

95. X. Zhang, L. Liu, T. Zhao, X. Chen, S. Lin, J. Wang, J. Mi, L. Wendi, GWL_FCS30: A global 30 m wetland map with a fine classification system using multi-sourced and time-series remote sensing imagery in 2020. *Earth Syst. Sci. Data* **15**, 265–293 (2023).
